# CAR^+^ and CAR^−^ T cells share a differentiation trajectory into an NK-like subset after CD19 CAR T cell infusion in patients with B cell malignancies

**DOI:** 10.1038/s41467-023-43656-7

**Published:** 2023-11-27

**Authors:** Raymond Hall Yip Louie, Curtis Cai, Jerome Samir, Mandeep Singh, Ira W. Deveson, James M. Ferguson, Timothy G. Amos, Helen Marie McGuire, Kavitha Gowrishankar, Thiruni Adikari, Robert Balderas, Martina Bonomi, Marco Ruella, David Bishop, David Gottlieb, Emily Blyth, Kenneth Micklethwaite, Fabio Luciani

**Affiliations:** 1 School of Computer Science and Engineering, UNSW Sydney, Sydney, NSW Australia; 2 Kirby Institute for Infection and Immunity, UNSW Sydney, Sydney, NSW Australia; 3 School of Medical Sciences, UNSW Sydney, Sydney, NSW Australia; 4 Garvan Institute for Medical Research, Sydney, NSW Australia; 5 Ramaciotti Facility for Human Systems Biology, The University of Sydney, Sydney, NSW Australia; 6 Charles Perkins Centre, The University of Sydney, Sydney, NSW Australia; 7 Infection, Immunity and Inflammation Theme, School of Medical Sciences, Faculty of Medicine and Health, The University of Sydney, Camperdown, NSW Australia; 8 Blood Transplant and Cell Therapies Program, Department of Haematology, Westmead Hospital, Sydney, NSW Australia; 9 Westmead Institute for Medical Research, Sydney, NSW Australia; 10 Becton Dickinson, San Jose, CA USA; 11 Department of Physics, University of Bologna, Bologna, Italy; 12 Division of Hematology and Oncology, University of Pennsylvania, Philadelphia, PA USA; 13 Sydney Medical School, The University of Sydney, Sydney, NSW Australia; 14 NSW Health Pathology Blood Transplant and Cell Therapies Laboratory – ICPMR Westmead, Sydney, NSW Australia

**Keywords:** Adaptive immunity, Immunosuppression

## Abstract

Chimeric antigen receptor (CAR) T cell therapy is effective in treating B cell malignancies, but factors influencing the persistence of functional CAR^+^ T cells, such as product composition, patients’ lymphodepletion, and immune reconstitution, are not well understood. To shed light on this issue, here we conduct a single-cell multi-omics analysis of transcriptional, clonal, and phenotypic profiles from pre- to 1-month post-infusion of CAR^+^ and CAR^−^ T cells from patients from a CARTELL study (ACTRN12617001579381) who received a donor-derived 4-1BB CAR product targeting CD19. Following infusion, CAR^+^ T cells and CAR^−^ T cells shows similar differentiation profiles with clonally expanded populations across heterogeneous phenotypes, demonstrating clonal lineages and phenotypic plasticity. We validate these findings in 31 patients with large B cell lymphoma treated with CD19 CAR T therapy. For these patients, we identify using longitudinal mass-cytometry data an association between NK-like subsets and clinical outcomes at 6 months with both CAR^+^ and CAR^−^ T cells. These results suggest that non-CAR-derived signals can provide information about patients’ immune recovery and be used as correlate of clinically relevant parameters.

## Abstract (web summary)

Phenotype of cells in the infusion product as well at specific post-infusion time points has been associated with clinical response to CD19 CAR T cells. Here the authors present a single-cell multi-omics analysis of pre- and post-infusion CAR+ and CAR- T cells from patients with relapsed or refractory B-ALL or LBCL who received CD19 CAR T therapy.

## Introduction

Chimeric antigen receptor (CAR) T cells engineered to recognise the CD19 protein are an effective cure for B cell malignancies such as diffused large B cell lymphoma, follicular lymphoma, mantle cell lymphoma, and B cell acute lymphoblastic leukaemia (B-ALL)^1–6^. Critically, long-term remission of at least 10 years is now a reality^7^. Treatment success however, requires the careful management of associated conditions including cytokine release syndrome (CRS), immune effector cell-associated neurotoxicity syndrome (ICAN), infection due to pre-infusion lymphodepleting chemotherapy, ensuring the persistence of functional CAR T cells^8,9^, as well as the quality of the CAR T cell infusion product (IP). Numerous studies have demonstrated that the phenotype of cells in the infusion product may be linked to the treatment response after infusion into patients. For example, a poor response was correlated with an exhausted phenotype^10^, while remission at 3-month follow-up had three-fold higher frequencies of endogenous CD8^+^ T cells expressing central memory signatures^11^. In a recent study, mesothelin-directed CAR T cells against pancreatic cancer developed a dysfunctional exhausted-like state with an NK-like phenotype after persistent antigen stimulation, which was confirmed in patients treated with CAR19 therapy, and were identified by transcription factors ID3 and SOX4^12^.

Currently, our understanding of how CAR T cells evolve at a cellular and molecular level following infusion and how this may be influenced by the patient’s immune system is limited. Recent studies have linked CAR T cell phenotypes at post-infusion time points with clinical outcomes. For example, the disease progression of large B cell lymphoma (LBCL) patients at six months could be predicted by the proportion of T regulatory (Tregs) phenotype in CAR19 T cells with CD28 costimulatory factor (Axi-cel) on the peak day^13^. Furthermore, this study also identified a subset of CAR^+^ T cells with an innate, natural killer (NK)-like phenotype that was associated with complete remission (defined as CD57^+^ cells, a phenotype also previously associated with terminal effector conventional or early senescent T cells^14^). In a separate study investigating LBCL patients treated with Axi-cel and Tisa-cel (CAR19 with 4-1BB costimulatory factor) products, CAR T cells from the two products had distinct molecular and phenotypic distributions, and increased fractions of Tregs with suppressive function in patients with poor outcomes^15^. Lastly, a recent study revealed that CAR^+^ T cells from non-responding patients overexpressed the exhaustion marker TIGIT at day 30 post-infusion when compared to those with complete or partial remission^16^.

Patients undergo immune reconstitution following hematopoietic stem cell transplantation (HSCT) because of lymphodepletion. Following infusion, T cells undergo rapid proliferation, forming predominantly effector T cells expressing CD57 and lacking CD28^17^, and with skewing of the CD4:CD8 T cell ratio towards the latter^18^. Assisted immune reconstitution with the adoptive cell transfer of vaccine-primed autologous cells in lymphopenic patients may minimise period of compromised immune function in a patient, but interestingly has been demonstrated to coincide with the clonal expansion of T cells specific for targets such as CMV which were not included in the product^19^. Limited knowledge exists on how lymphodepletion and subsequent immune reconstitution may shape the molecular and phenotypic characteristics of CAR T cells and ultimately CAR T cell therapy outcome. Treatment with cyclophosphamide/fludarabine prior to cell infusion has been associated with CAR T cell persistence in patients with acute lymphoblastic leukaemia^20,21^.

Clonal expansion of CAR^+^ T cells following infusion results in large clones with effector and terminally differentiated phenotypes^13,22^. These clones may be preferentially expanded through the additive signalling provided by endogenous TCRs engaging with cognate antigens^23^. Whilst donor and recipients were matched on the basis of major HLA types, it is feasible that mismatched minor HLA types and other presentation pathways may interfere with CAR T cell functions through aberrant signalling involving endogenous NK and Tγδ cells^24^. These unconventional immune cells play key roles in the control of infections^25^, provide anti-tumour responses independent of MHC molecules^26^, and regulate allogenic responses following HSCT^27^. Determining how the endogenous immune cells from patients may shape infused CAR^+^ T cells, could also lead to the discovery of shared differentiation dynamics between these two compartments and an improved understanding of the precise mechanisms through which CAR T cells differentiate and acquire their specific phenotypes long after initial infusion.

In this study, we analyse eight patients who received donor-derived piggyBac-modified CD19 CAR T cells^28^. We examine both circulating immune cells and CAR^+^ T cells using a combination of single-cell RNA sequencing, protein expression profiling, and T cell receptor sequencing, as well as mass-cytometry analysis of pre- and post-infusion blood samples. Our findings indicate that CAR^+^ and CAR^−^ CD8^+^ T cells share a differentiation trajectory that is influenced by endogenous pathways such as TCR signalling and homoeostatic proliferation. We validate these results on a larger cohort of patients^13^ and identify correlations between the proportions of CAR^+^ and CAR^−^ NK-like and regulatory CD8 T cell subsets with clinical outcomes. Our study provides molecular and phenotypic evidence to describe pathways of T cell differentiation that are shared between CAR^+^ and CAR^−^ cells following CAR19 T cell infusion and identifies cell subsets that can be further explored to identify correlates of therapy outcome and improve future therapies.

## Results

### Single-cell atlas of PBMC and CAR T cells reveals post-infusion expansion of CD8^+^ T cells

**Fig. 1:**
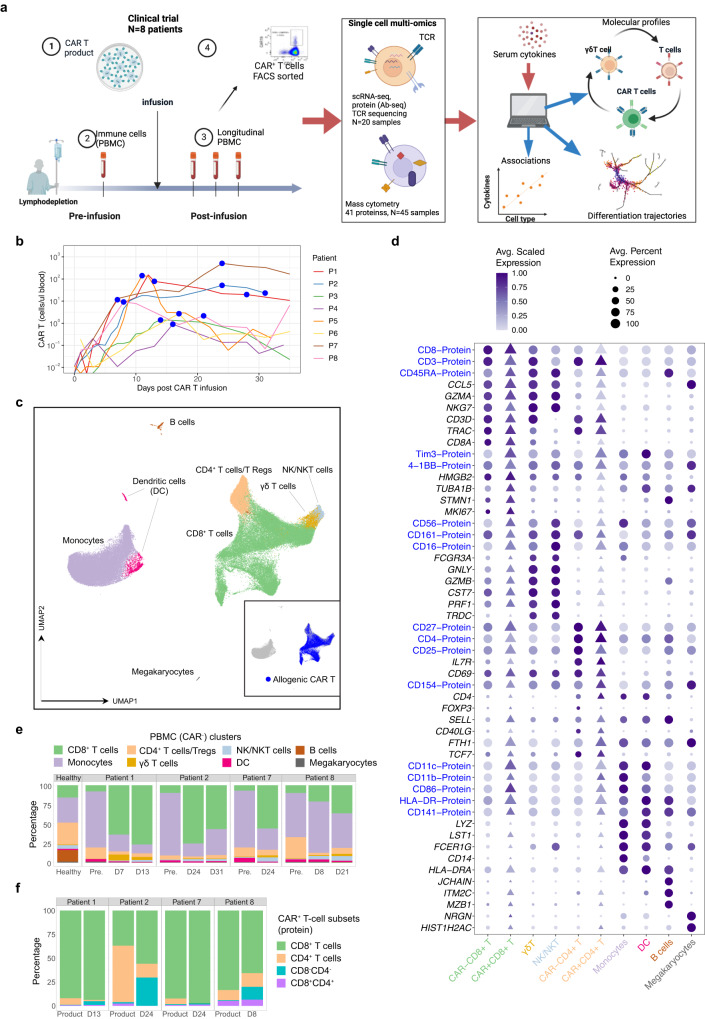
Longitudinal analysis of the molecular and cellular composition of allogenic CAR T and endogenous patient immune cells from pre- to post-infusion. **a** Overview of study design (created with Biorender.com). For each patient (*N* *=* 8), peripheral blood mononuclear cells (PBMC) from pre- and post-infusion samples, and CAR T cells from product and post-infusion samples were analysed. Single-cell multi-omics (*N* *=* 4) and mass cytometry (*N* *=* 8) were utilised to generate molecular profiles of immune cells, and these were then used to identify differentiation trajectories and associations with cytokines. **b** Kinetics of CAR T cells following infusion, blue symbols indicate sample time points utilised for downstream multi-omics. **c** Dimensionality reduction using UMAP of CAR^+^ and CAR^−^ T cells using single-cell transcriptomics showing canonical cell types. **d** Dot plot of selected proteins and genes. The size of each point represents the percentage of cells with non-zero expression. Colours represent the scaled log expression averaged over all endogenous or CAR T samples. CAR^−^ cells are represented by filled circles, CAR^+^ by filled triangles. **e** Distribution of canonical cell types in the CAR^−^ samples. **f** Distribution of CD8^+^ and CD4^+^ subsets in the CAR^+^ samples.

We analysed eight patients (Supplementary Table 1) with relapsed or refractory B-ALL or LBCL following HSCT who were treated with piggyBac-modified CD19 CAR T cells (second generation) within a clinical trial^28,29^. Patients underwent combined cyclophosphamide and fludarabine lymphodepletion conditioning for three days prior to infusion (Fig. 1a). Seven patients (P1, P2, P3, P4, P5, P7, P8) received allogenic CART19 generated from cells obtained from the same sibling HSCT donor and one patient (P6) received an autologous CAR T transfer. All patients had haemopoietic reconstitution after HCST and before CAR T cell infusion except for P3 who had partial donor bone marrow chimerism. Robust expansion of CAR T cells was observed with peak count typically occurring by the third week (Fig. 1b).

To understand the reconstitution of the patients’ immune populations, and of CAR T cells during the initial stage of therapy (Fig. 1a), we sampled from peripheral blood mononuclear cells (PBMC) one day before infusion and longitudinally up to a month post-infusion. CAR T cells were obtained from the IP and after infusion using flow cytometric sorting for CD3^+^CAR^+^ cells from PBMC (Fig. 1a, Supplementary Fig. 1a) and we further validated the presence of CAR^+^ T cells within the total sequenced PBMC populations by detection of the CAR transcript (Supplementary Fig. 1b). Hereafter, analyses referring to CAR^+^ cells include those sorted using an anti-CAR antibody or identified by positive expression of the CAR transcript, while CAR^−^ cells were those from the PBMC fraction lacking the CAR transcript. We analysed 106,976 cells from four patients (P1, P2, P7 and P8), and measured the expression of genes and of 41 extracellular proteins, and full-length T cell receptor (TCR) sequence across 20 samples (11 PBMC, 8 CAR T cell sorted populations, 1 publicly available healthy donor PBMC) (Supplementary Tables 2 and 3). Furthermore, using a panel of 41 protein markers in mass cytometry (CyTOF), we characterised the protein profiles of PBMC and CAR T cells across all eight patients (Supplementary Table 4, Fig. 1a).

We performed dimensionality reduction and clustering based on gene expression profiles and identified eight clusters corresponding to canonical cell types (Fig. 1c, d). These comprised of CD8^+^ T cells, CD4^+^ Treg CD25^+^*FOXP3*^+^ cells, NK/NKT, Tγδ cells, B cells, dendritic cells, megakaryocytes, and monocytes (Fig. 1c). All patients shared common profiles of gene and protein expression (Supplementary Fig. 1c, Supplementary Data 1) and altered subset distributions exemplified by a reduced proportion of monocytes at post-infusion when compared to pre-infusion time points (Fig. 1e). Conversely, the proportion of CAR^−^CD8^+^ T cells and Tγδ cells was increased at post-infusion time points as compared to baseline (Fig. 1e, Supplementary Fig. 1f, g). As expected, very few B cells avoided CAR T cell killing in the blood at post-infusion time points. The largest cluster comprised CD8^+^ T cells including both CAR^−^ (Fig. 1e) and CAR^+^ (Fig. 1f) cells, which was consistent with CD8^+^ being the most common T cell subset as measured by mass cytometry (Supplementary Fig. 1e, f). More specifically in our single-cell multi-omics dataset, the majority of CAR^+^ T cells were found in the CD8^+^ subset except for the IP in P2, P4 and P6 which were majority CD4^+^ (Fig. 1f and Supplementary Fig. 1f). Both CAR^+^CD8^+^ and CAR^−^CD8^+^ T cells expressed intermediate levels of effector (*GZMA*, *PRF1*, *IL32*) and NK-like markers (CD56, CD16, CD161, *NKG7, FCGRA*) (Fig. 1d, Supplementary Fig. 1g), suggesting that both populations included cytotoxic NK-like (innate-like) CD8^+^ T cells^30–32^. In summary, single-cell multi-omics and mass cytometry revealed significant expansion of CD8^*+*^ and Tγδ cells following infusion, with CAR^+^ and CAR^−^ T cells sharing similar molecular profiles.

### CAR^−^ and CAR^+^ T cells form distinct subsets including NK-like phenotype

**Fig. 2:**
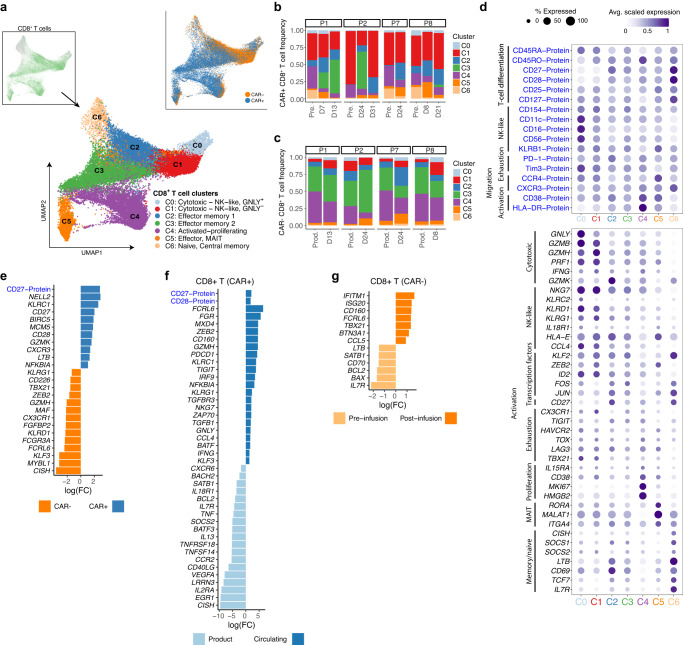
Single-cell multi-omics identify heterogeneous subsets of CD8^+^ T cells shared between CAR^−^ and CAR^+^ T cells. **a** Dimensionality reduction (UMAP) of CD8^*+*^ T cells from both CAR^+^ and CAR^−^ samples using scRNA-seq data, revealing six clusters. Top left inlet plot shows the position of the CD8 clusters in the larger UMAP plot, top right, the distribution of CAR^+^ and CAR^−^ CD8^+^ T cells. **b**, **c** Distribution of the clusters by sample time point and patient for both CAR^−^ (**b**) and CAR^+^ T cells (**c**). **d** Dot plots of expression values for selected genes and proteins (AbSeq). Shown are gene markers with significant *p*-value (<0.1 using MAST algorithm, two-sided *t*-test with multiple-hypothesis testing). The size of each point represents the percentage of cells with non-zero expression. The colour represents the scaled log expression averaged over all CAR^−^ or CAR^+^ T samples. **e** Bar plots showing fold changes (log-FC) from a set of representative genes and proteins (see Supplementary for full list). Fold-change values for gene and proteins were obtained from differential expression analysis between CAR^+^ and CAR^−^ CD8^+^ T cells circulating in post-infusion samples. Differential expression analysis was performed using *N* = 4 patients as replicates (adjusted *p*-value < 0.05, edgeR two-sided test). **f**, **g** Same as (**e**), differential expression analyses between Circulating and Infused CAR^+^ T cells (**f**), and between pre- and post-infusion CAR^−^ T cells (**g**). Data are from 4 patients across 20 sample time points.

We further investigated the heterogeneity and differentiation dynamics of the CD8^*+*^ T cell population, as this was the predominant subset in the blood following infusion. The CD8^+^ T cell population classified into seven clusters (C0-C6) (Fig. 2a, Supplementary Fig. 2a–c) with 44.5% (*N* *=* 7440/16719) of total CAR^−^CD8^+^ T cells found in C1 (Fig. 2b) and 60% (*N* *=* 13,595/22,291) of the CAR^+^CD8^+^ T cells in C3 and C4 (Fig. 2c).

Interestingly, two clusters, C0 and C1, identified NK-like T cells, and expressed NK-like protein markers CD56, CD16, CD11c, and KLRB1 (Fig. 2d, Supplementary Data 1), and genes *NKG7, KLRG1, KLRD1*, and *IL18R1* at comparable levels to those expressed by NK and NKT cells (Supplementary Fig. 1d). C0 revealed a higher expression of NK-like signature than C1, including *KLRC2* (encoding for NKG2C), and having higher expression of NK-like protein markers and Tim-3. This NK-like profile in CD8^+^ T cells has been previously observed in viral infection^33^ and cancer^30^, as well as in CAR T cells under persisting stimulation in vitro^12^.

The neighbouring clusters C2 and C3 had an effector-memory phenotype and expressed intermediate levels of activation markers (CD38, HLA-DR, PD-1). Both clusters C2 and C3 contained a subset of cells expressing AP-1 transcription factors, e.g., *FOS* and *JUN*, which are known to be associated with resistance to exhaustion in CAR T cells^34^, and a lack of these factors has been found to be indicative of progenitor of exhausted T cells^35^. Gene Set Enrichment Analysis (GSEA) (Supplementary Fig. 2d, Supplementary Data 2) also revealed enrichment for a curated progenitor of exhaustion gene set but not for terminal exhaustion.

Cells within C4 also expressed the same activation markers, as well as markers of proliferation (*MKI67, HMGB2*), and exhaustion (Tim-3, PD-1), and genes related to development of the exhausted phenotype (*TOX*, *TIGIT*, *CX3CR1*)^10,36,37^ (Fig. 2d). This profile, together with a cell-cycle state in G2M and S phases (Supplementary Fig. 2c) resembled the highly proliferative dysfunctional subset previously identified within tumour-infiltrating CD8^+^ T cells in melanoma^36^. Cluster C5 also revealed an effector profile, consistent with unconventional mucosal associated invariant T (MAIT) cells expressing KLRB1 (CD161), CD38 and CCR4 proteins, and genes *IL18R1* and *CXCR4*^38^ (Fig. 2d). Finally, naïve and central memory cells formed cluster C6, expressing proteins CD27, CD28, CCR7, and CD127, and genes such as transcription factors *TCF7, IL7R*, and AP-1 transcription factors *FOS* and *JUND* (Fig. 2d).

In summary, this analysis showed heterogeneous subsets of CD8 T cells comprising both CAR^+^ and CAR^−^ T cells, and notably with CAR^−^ T cell dominating the NK-like clusters, while CAR^+^ T cells comprised mostly of effector and proliferating subsets.

### Following infusion, CAR^+^ and CAR^−^ T cells differ in the extent of differentiation and NK-like profile, with CAR^+^ rapidly differentiating into effector and proliferating subsets

We reasoned that whilst CAR^+^ and CAR^−^ T cells clustered together and broadly shared molecular and phenotypic changes, there may be more subtle differences between CAR^+^ and CAR^−^ T cells as they differentiated from pre- to post- infusion time points.

Pairwise comparison between CAR^+^ and CAR^−^ T cells at post-infusion showed that CAR^+^ T cells up-regulated genes associated with NK-like responses (*KLRC1*), cytotoxicity (*GZMK* and *LTB*), and proliferation (*MKI67*)^37,39^ (Fig. 2e), and down-regulated genes associated with the suppression of cytokine signalling (*SOCS2* and *CISH*) (Supplementary Data 1). By contrast, CAR^−^ T cells up-regulated another cytotoxic gene (*GZMH*), markers characterised to restrict cytotoxicity (*KLRD1*, *KLRG1*)^40^, and transcription factors associated with promoting terminal differentiation (*KLF3*, *ZEB2*)^41^. GSEA confirmed these results, with circulating CAR^+^ T cells enriched for exhaustion and metabolic signature scores (oxidative phosphorylation and MYC targets), while CAR^−^ T cells were enriched for NK-like and regulatory functions mediated by TNFα signalling (Supplementary Data 2).

Extensive differences were detected between CAR^+^CD8^+^ T cells circulating in the blood and product cells (4960 differential expressed genes, adjusted *p*-value < 0.05, Fig. 2f and Supplementary Data 1). Circulating CAR^+^ T cells were enriched for cytotoxic, NK-like markers (*IFNG, KLRC1, KLRG1, FCRL6, CD160)*, TCR signalling (*ZAP70*), exhaustion (*PDCD1, TIGIT*, *TOX*)^42,43^, and genes associated with regulating effector functions (*IRF9* and *TGFB*)^44,45^. They up-regulated transcription factors *ZEB2* and *MXD4*, which are negative regulators of MYC, and both are known to favour effector T cell differentiation^46,47^. In contrast, product cells expressed *SOCS2* and *CISH*, thus suggesting active silencing of TCR signalling^48^, and also up-regulated *IL2RA* (CD25), *IL7R*, and *IL18R1*, and transcription factors *EGR1*, and *BACH2*^49^, which suggests interleukin-induced proliferation.

These results suggest that following infusion, CAR^+^ T cells rapidly acquire an innate-like/effector/killing phenotype. Interestingly, the CAR^−^ compartment shows fewer differences when compared with pre-infusion cells (418 genes, adjusted *p*-value < 0.05, Supplementary Data 1), showing increased NK-like activating markers (*FCRL6, TBX21*, and *CD160*) and IFN-γ pathway genes *ISG20* and *IFITM1* (Fig. 2g). These results hence suggest that additional signalling received by the CAR construct seeing CD19 may act as additional stimulus towards more rapid proliferation and differentiation.

### Validation of the NK-like signatures using published single-cell multi data

To validate the molecular signatures identified within the cytotoxic and NK-like cells of clusters C0 and C1, we performed reference-based clustering on two published scRNA-seq datasets of CAR^+^ and CAR^−^ CD8^+^ T cells and tested the hypothesis that the CD8^+^ T cells from these two cohorts can be classified into the 7 subsets identified in our study. The first dataset consisted of longitudinal samples from pre-infusion to day 7 post-infusion from 32 patients with large B cell lymphoma treated with Axi-cel (*N* *=* 19) or Tisa-cel (*N* *=* 13)^15^. We confirmed that CD8^+^ CAR^+^ and CAR^−^ T cells divide into all 7 subsets, including the cytotoxic, NK-like signatures from C0 and C1. This analysis also confirmed the published results from these data, in that Tisa-cel products were predominantly formed by CAR^+^ T cells with a proliferating memory signature, which correspond to our cluster C4, while Axi-cel consisted of a more heterogeneous distribution of subsets (Supplementary Fig. 3a). We found a significant increase of cells in C0 and C1 subset in post-infusion samples compared to product, and for both Axi-cel and Tisa-cel (Supplementary Fig. 3c), in line with our observation from the CARTELL cohort. Importantly, gene expression analysis identified that the NK-like cells from C0 and C1 had a gene signature consistent with those identified in the CARTELL cohort (Supplementary Fig. 3d), for instance the expression of *FCGRA* (encoding CD16), *NKG7*, *CCL4*, *KLRD1*, and *KLRC1* encoding the C-type lectin-like receptor superfamily NKG2A (Supplementary Data 1). The second dataset of CAR^+^ T cells were scRNA-seq data of product and post-infusion samples from a paediatric cohort of 15 patients treated with autologous 4-1BB CART19^22^ (Supplementary Fig. 3e, f). Reference-based clustering on these data confirmed the existence of CD8^+^ T cell subsets with a gene signature consistent with those predicted in C0 and C1. Notably, only cells from patients that responded to therapy (*N* *=* 12) were found in C0 and C1, however the small sample size prevented statistical significance analysis. These results demonstrate that the cell distribution identified with our comprehensive single-cell multi-omics is robust across a larger cohort of patients and supports the validity of identifying subsets of innate-like cells in three distinct CAR therapies.

### CAR^+^ and CAR^−^ T cells undergo clonal expansion and demonstrate intra-clonal cellular heterogeneity

T cell clones identified as cells with identical endogenous TCRαβ CDR3 sequences act as a proxy for identifying potential T cell lineages and investigating the mechanisms underlying their differentiation. Therefore, we recovered full-length TCR sequences of T cells from our longitudinal samples to infer T cell lineages and intra-clonal plasticity that may occur during pre- to post-infusion differentiation^50^.

**Fig. 3:**
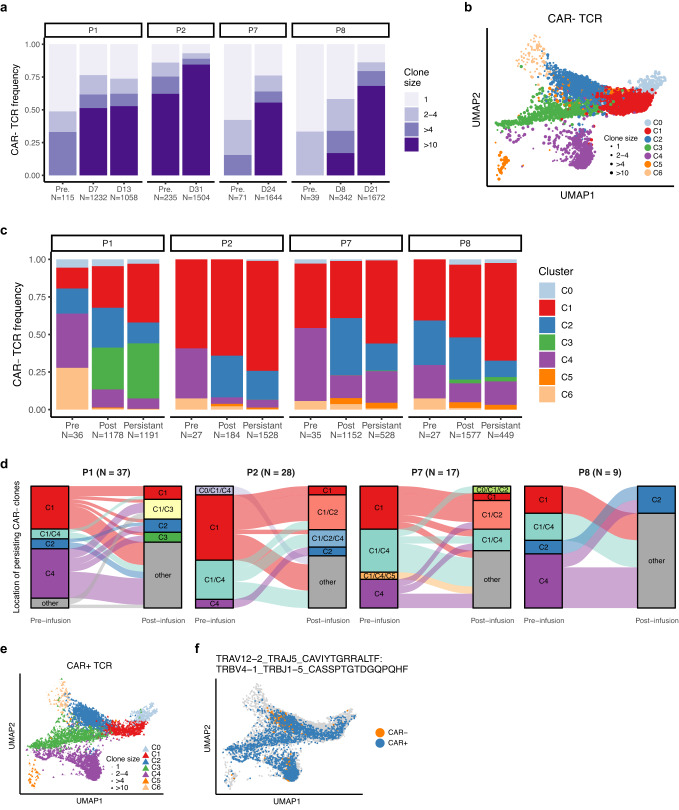
Clonal analyses reveal plasticity in differentiation and fate of CAR^−^ and CAR^+^ CD8^+^ T cells during therapy. **a** Distribution of clone size in CAR^−^CD8^+^ T cells in 4 patients and time points. Colours represent group size from singletons to clones with size > 10. **b** Post-infusion expanded clones (size > 1) identified in CAR^−^ cells, mapped on the UMAP plot. **c** Percentages of CAR^−^ CD8^+^ clones in each sample time point. Colour represents clusters. Number of clones are shown in the legend of the x-axis. **d** Alluvial plots showing the distribution of persisting clones (lineages) from pre- to post-infusion samples, according to the cell clusters identified via the UMAP analysis. Each line represents an individual lineage. The box “other” represents combinations of 3 or more clusters where the clone was identified, and were grouped together for the sake of readability. The number of lineages is shown for each patient. **e** Post-infusion expanded clones (size > 1) identified in CAR^+^ cells, mapped on the UMAP plot. **f** Mapping of a single clone with a TCRαβ that matched a TCR sequence identified in P7, showing heterogeneous distribution across all clusters and in both CAR^+^ and CAR^−^ T cells.

There were 1914 and 1231 clones identified in CAR^−^ CD8^+^ and CAR^+^ CD8^+^ T cells, respectively (Supplementary Data 3). CAR^−^CD8^+^ T cells showed increased clonal expansion from pre- to post-infusion (Fig. 3a). Clonal expansion primarily occurred within cytotoxic subsets (C1) in post-infusion samples, and additionally in the highly proliferative subset (C4) in pre-infusion samples (Fig. 3b). Furthermore, 47% (3696/7912) of the CAR^−^CD8^+^ T cells included clones (*N* *=* 91) which persisted following infusion and were found to be present in all clusters (Fig. 3c), thus suggesting intra-clonal plasticity during differentiation from pre- to post-infusion (Fig. 3d). Notably, there were 96 clones from cells found in C0 which were also found in cells from other clusters, including memory phenotype, thus suggesting that NK-like T cells differentiated from pre-existing CD8^+^ T cells (Supplementary Fig. 4a). Before infusion, the clones that persisted during therapy were primarily distributed across clusters and mostly in C1 and C4. However, following infusion, there was a phenotypic shift towards cytotoxic and effector subsets (C1 and C2) (Fig. 3d), indicating differentiation within NK-like lineages during therapy. CAR^+^CD8^+^ T cells formed a more diverse clonal repertoire compared to CAR^−^ T cells, and large clones of size > 10 were found only in one patient (Fig. 3e, Supplementary Fig. 4b). This patient (P7) had a single large clone comprising 59% (2865/4822) of the total CAR^+^ T cell repertoire, and carried a TCR without known epitope specificity (when cross-referencing with VDJdb^51^). The same clone was also detected in 15% (252/1715) of the CAR^−^ T cells from the same patient, and it was not found at pre-infusion, thus suggesting that CAR^−^ T cells within this clone could be derived from the infusion product or low frequency undetected patient’s cells. Only a small proportion of cells with clonotypes shared between CAR^+^ and CAR^−^ cells were detected in the other three patients.

We queried VDJdb^51^ to identify the epitope specificity of TCR sequences identified in CAR^−^ and CAR^+^ T cells and found only 9 clones with an exact match (in at least one chain, α or β, Supplementary Data 4) to sequences specific for CMV epitope, while 472 TCR (15% of total) had a close distance (maximum of 3 mismatches in either chain) to TCR specific for known epitopes (predominantly CMV, influenza, and EBV^51^) (Supplementary Data 4, Supplementary Fig. 4c). In all 4 patients, TCR sequences with a close distance to known specificities were found mostly within large clones (size > 4) and were detected in cells from both pre- and post-infusion samples (Supplementary Fig. 4d, e). All patients, except P5 were CMV sero-positive although no clinically relevant viral reactivation was observed in any patients during the first month post-infusion.

### Lineage tracing of CAR^−^ and CAR^+^ CD8^+^ T cells demonstrate a shared differentiation trajectory terminating into NK-like state in post-infusion samples

**Fig. 4:**
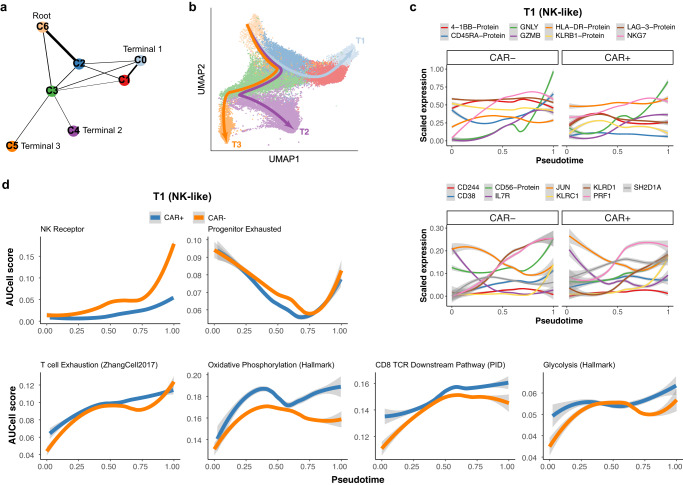
Trajectory analysis reveal terminal states of both CAR^+^ and CAR^−^ T cells during therapy. **a** PAGA graph obtained from CAR^−^ and CAR^+^ CD8^+^ T cells, revealing cluster connectivity (represented by line weights) and 3 terminal cell states. **b** Pseudotime values and 3 inferred trajectories obtained by assuming a random cell in cluster C6 as root. Trajectories were generated using Slingshot (see the “Methods” section). **c** Both panels show the genes and surface protein expression along the inferred trajectory T1 leading to a terminal NK-like state. The scaled expression values are plotted against the inferred pseudotime values, lines represent the inferred loess curves. Data are presented as a smoothed curve using weighted polynomials around neighbouring point, with grey shade representing a 95% confidence interval. **d** Same as panel (**c**), shown are the inferred gene signature scores estimated using AUCell along the inferred pseudotime values in T1.

We used the property of dividing T cells to retain their endogenous TCR sequence to track clones between pre- to post-infusion samples to infer T cell lineages and intra-clonal plasticity that may occur during differentiation. To understand the differentiation process across cell states, we used a probabilistic graph-based approach (PAGA)^52^ to quantify the inter-cluster connectivity (Fig. 4a). We observed connections between the naïve/central memory cluster, C6, and the effector-memory cluster, C2, and the cytotoxic NK-like clusters (C0 and C1), confirming the clonal expansion data. Given these connections we applied pseudotime trajectory analysis choosing a random cell in C6 as a root, and investigating three differentiation trajectories, each identifying a transition towards a terminal cell state: T1 – NK-like state (C0), T2 – activating-proliferating state (C4), and finally T5 – effector-MAIT (C5) (Fig. 4b). Along T1 both CAR^+^ and CAR^−^ T cell subsets were characterised by increasing expression of TCR signalling, cytotoxicity (*GZMB* and *PRF1*), and NK-like (*GNLY*, *NKG7, KLRC1* and CD56 protein) signatures (Fig. 4c, d). In contrast, declining trends were observed for AP-1 transcription factors (*FOS* and *JUN*), memory (*IL7R*), and signature score of progenitors of exhausted cells. Higher levels of exhaustion, glycolysis and oxidative phosphorylation were obtained towards the terminal state (Fig. 4d).

The trajectories, T2 and T3, both showed a decline in cytotoxicity, NK-like profile and TCR signalling towards the terminal cell states, i.e., activating-proliferating (C4) and effector-MAIT (C5), respectively. T2 showed increase in exhaustion and oxidative phosphorylation and glycolysis pathways, as well as proliferation associated genes (e.g., *MKI67*) (Supplementary Fig. 5a–d). In contrast, cells along T3 showed an increase in activation markers, e.g., HLA-DR and of MAIT markers (*RORA*, *MALAT1*).

Finally, we analysed the subset of circulating CAR^−^ T cells, which persisted from pre-infusion and that shared a TCR sequence, hence providing a proxy for the true patient-derived CAR^−^ T cell lineages. These persisting clones were distributed across all clusters and formed a similar differentiation trajectory to those estimated from the total CAR^−^ T cell subsets (Supplementary Fig. 5e, f). These data support the conclusion that CAR^−^ T cells follow a similar differentiation trajectory regardless of their origin (patient or product).

### Clonal analysis of the patients who later developed CAR T cell-induced lymphoma

To test whether the malignant clones identified in patients P2 and P8 influenced the clonal analysis in all 4 patients, we investigated the endogenous TCR repertoire of patients P2 and P8, who developed CAR T cell-induced lymphoma diagnosed after a year post-infusion. The two CAR T cell-induced lymphomas were each identified as a monoclonal population of CAR^+^ T cells, with P2 developing a malignant CD4^+^CD3^+^ clone with sequence TRAV30*01 TRAJ5*01 TRBV2*01 TRBD*01 TRBJ1-3*01 (CGTIDTGRRALTF- CASSTQGSGNTIYF), and P8 with a malignant CD8^+^ clone with TRAV9-2_TRAJ28/TRBV7-8_TRBJ1-5 (CAPIYSGAGSYQLTF-CASSSPDRGENQPQHF). Deep TCRβ sequencing of the CD4 T cells in the infusion product of P2 showed a diverse repertoire, with the malignant clone in only 1/1436 sequences, and for P8 the malignant clone was not detectable in the 13,937 TCRβ sequences from the CD8^+^ infusion product cells. Analysis of the CD8^+^ TCR repertoire in post-infusion samples found the malignant clone in 1/240 CD8^+^CAR^+^ at day 8 and 0 in 609 CAR^−^ at day 8, and 3 of 2657 CD8^+^CAR^−^ cells at Day 21 in P8, respectively. In P2, the malignant clone was found in 11 of 354 (3%) CAR^+^ T cells at day 24 and in 3 of 2877 of the T cells identified from the patient’s PBMC (c). For P2, additional single-cell plate-based sorting of CAR^+^ T cells using flow cytometry and Smartseq2 protocol^53^ detected the malignant clone in 3/47 (6%) cells at day 31. These results suggest that the malignant clones are present in a small proportion of the CAR^+^ T cells and are unlikely to influence the differentiation and expansion kinetics of CAR^+^ cells.

### Validation of the cell heterogeneity and NK-like profiles from novel and published proteomics data

**Fig. 5:**
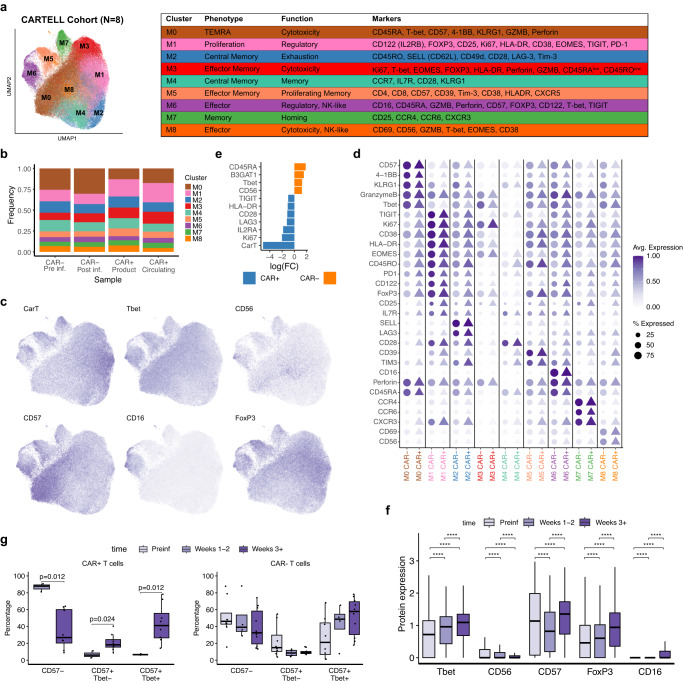
Mass cytometry from the CARTELL cohort confirmed a shared protein profiles in CAR^−^ and CAR^+^ CD8^+^ cells and increase of NK-like T cell subsets following infusion. **a** Dimensionality reduction and clustering using UMAP of both CAR^+^ and CAR^−^ CD8^*+*^ T cells identifying 9 clusters. Data are from the 8 patients in the CARTELL cohort. **b** Distribution of CARTELL samples in each identified cluster. **c** Selected protein expression plotted over UMAP. Darker colours represent greater expression. **d** Dot plot of scaled protein expression values for both CAR^+^ and CAR^−^ T cells in each cluster identified from the CARTELL samples. The size of each point represents the percentage of cells with non-zero expression. The colour represents the avg. (average) scaled log expression. **e** Differential protein expression showing differences between CAR^+^ and CAR^−^ T cells (*N* = 8). Shows are markers with adjusted *p*-value < 0.05, using edgeR (two-sided). **f** Selected protein expression values over time of CAR^+^ and CAR^−^ CD8^+^ T cells ^(^*N* = 8). Cells from the infusion product are removed. Adjusted *p*-values (paired two-sided Wilcox test with Bonferroni correction between time points) are shown and abbreviated to significance stars (****p < 0.0001) for readability. **g** Proportion of CD8^+^ subsets defined by CD57/T-bet expression in samples grouped by time point and split by CAR^+^ and CAR^−^ using *N* = 8 independent patients. All significant (*p* < 0.05) adjusted *p*-values (paired Wilcox test with Bonferroni correction between time points) are reported. Box plot whiskers extends to the minimum and maximum values, with the box encompassing the interquartile range (IQR), and the centre line indicating the median.

We next validated the major findings from the single-cell multi-omics data, using the longitudinal mass-cytometry data from all eight patients from the CARTELL trial. UMAP dimensionality reduction and shared nearest neighbour clustering confirmed the presence of phenotypically distinct subsets of CD8^+^ T cells, and specifically the presence of NK-like subsets. CD8^+^ T cells were classified into nine clusters (Fig. 5a), with a relatively homogeneous distribution of CAR^+^ and CAR^−^ cells, as well as pre- and post-infusion sample time points (Fig. 5b). Of note, two clusters were identified as having a high expression of NK-like markers, M6 contained effector cells expressing CD16 as well as canonical markers of cytotoxic CD8^+^ cells, e.g. GZMB and perforin, while M8 comprised of cells expressing CD56, as well as activation markers CD38 and CD69 (Fig. 5c, d). Cluster M1 was formed by proliferating cells, with elevated expression of Ki67, as well as exhaustion markers PD-1, TIGIT, and Eomes. Notably, this cluster also showed high level of FOXP3, CD49d, and CD122 (IL2RB), thus suggesting regulatory phenotype, which has been previously reported for suppressing allograft rejection for CD8 T cells^54,55^. M0, M6 and M8 consisted of effector cells with both a cytotoxic and an NK-like phenotype: M0 were T_EMRA_, expressing CD57, T-bet, and cytotoxic markers Granzyme B, Perforin, and 4-1BB (CD137). Cells in M2, M4, and M7 showed a memory profile, with M7 enriched for homing chemokines CCR4, CCR6 and CXCR3. Cluster M3 and M5 had effector-memory profiles, with M3 cells expressing proliferation markers (Ki67), while M5 had expression of both CD8 and CD4, and expressed FOXP3 and exhaustion markers CD39 and Tim-3. Differential protein expression analysis between CAR^+^ and CAR^−^ T cells in circulation showed that CAR^+^ T cells had higher level of Ki67, CD25, CD28 and LAG3, which is consistent with a more activated and differentiated status when compared to CAR^−^ T cells, which in contrast had increased CD57, T-bet and CD45RA (Fig. 5e). Longitudinal analysis of the CARTELL cohort showed increased expression of CD57 and T-bet, as well as of CD16 and FOXP3 from early phase (week 0 to 3) to late phase (weeks > 3) (Fig. 5f). Furthermore, within the CAR^+^ CD8^+^ T cells, both CD57^+^Tbet^+^ and CD57^+^Tbet^−^ populations increased over time (Fig. 5g), while for CAR^−^, the increase was observed but was not significant.

**Fig. 6:**
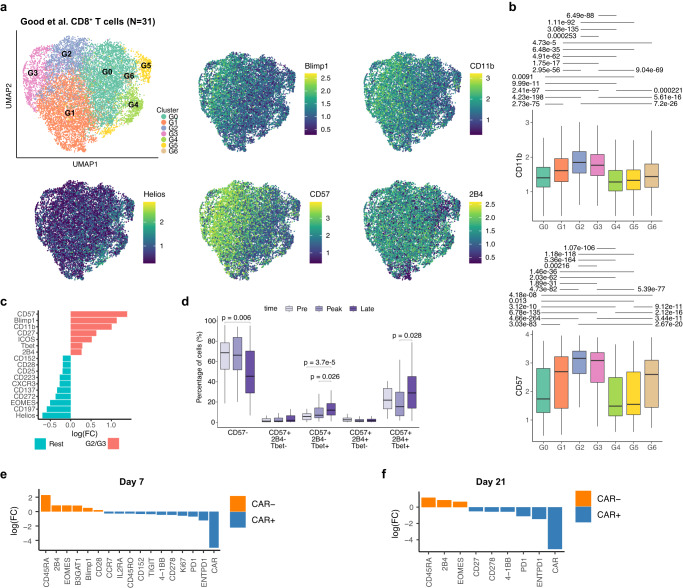
Validation cohort mass-cytometry confirmed the heterogeneity and shared protein profiles in CAR^−^ and CAR^+^ CD8^+^ cells. **a** Dimensionality reduction and clustering using UMAP of both CAR^+^ and CAR^−^ CD8^*+*^ T cells identifying 7 clusters using published data from 31 patients treated using Axi-cel. Also shown are five selected protein markers. **b** Box plots of CD57 and CD11b protein expression across clusters (*N* = 31). All significant (*p* < 0.05) adjusted *p*-values (paired Wilcox test with Bonferroni correction) are shown. **c** Differential protein expression showing differences between cells from G2 and G3 grouped together, and cells from other clusters (Rest). All markers shown have adjusted *p*-values < 0.05 (using edgeR, two-sided test). **d** Longitudinal analysis of the proportion of cells expressing CD57, T-bet and 2B4 over the course of therapy (*N* *=* 31). All significant (*p* < 0.05) adjusted *p*-values (paired Wilcox test with Bonferroni correction between time points) are reported. **e**, **f**. Differential protein expression showing differences between CAR^+^ and CAR^−^ T cells at day 7 (**e**) and day 21 (**f**). All markers shown have adjusted *p*-values < 0.05 (using edgeR, two-sided test). Box plot whiskers extends to the minimum and maximum values, with the box encompassing the interquartile range (IQR), and the centre line indicating the median.

To confirm these results, we analysed CD8^+^ T cells from the published mass-cytometry data from *N* = 31 patients with large B cell lymphoma undergoing CAR19 therapy with Axi-cel^13^. We performed unsupervised analysis on both CAR^+^ and CAR^−^ CD8 T cells using 34 protein markers and using pre- and post-infusion samples (Fig. 6). Dimensionality reduction and clustering revealed 7 subsets, which at large confirmed the major findings in the original article, showing segregation based on the expression of CD57 and T-bet, (Fig. 6a). These clusters were consistent with the subsets identified in the CARTELL cohort and notably a subset with high expression of CD57 and CD11b, which is an integrin protein found in NK cells and in activated CD8^+^ T cells (clusters G2 and G3, Fig. 6b), confirming the presence of a highly effector polarised phenotypes, as well as regulatory CD8 T cells expressing Helios (G5 and G6). Differential protein expression analysis showed that cells in G2 and G3 had increased expression of CD57, CD11b and Blimp, and lower levels of Helios and EOMES (Fig. 6c). Longitudinal analysis of the data showed an increase from pre-infusion to day 7 and then day 21 of the proportion of cells expressing CD57 and T-bet but lacking 2B4, which is a natural killer cell receptor found in NK and CD8^+^ cells^56^ (Fig. 6d), thus suggesting heterogeneous and varying subsets of CD8^+^ T cells with unconventional T cell profiles following infusion. Differential protein expression between CAR^+^ and CAR^−^ T cells at both day 7 and day 21 showed that CAR^−^ T cells were enriched for NK-like markers CD45RA, Eomes, CD57 and 2B4, while in contrast CAR^+^ T cells expressed higher level of Ki67, and of exhaustion markers CD39, PD-1 (Fig. 6e, f).

In summary, mass-cytometry data from our and the validation cohorts confirmed the shared molecular and phenotypic profiles between CAR^+^ and CAR^−^ T cells which was identified with single-cell multi-omics, and confirmed the increased NK-like or suppressive phenotypes on CAR^−^ T cells while CAR^+^ T cells showed increased cellular activation and exhaustion.

### Associations of CAR^+^ and CAR^−^ T cell subsets with clinical outcomes

Our results so far demonstrated that the molecular and cellular composition of both patient’s and CAR^+^ T cell during the early phase post-infusion identify the cell states through which these cells evolve. We reasoned that this information may be informative of therapy outcome, as it quantifies the perturbed immune-state of the patient. We investigated whether subsets of CD8^+^ T cells with an NK-like phenotype were associated with clinically relevant outcomes using longitudinal mass-cytometry data from the Axi-cel cohort^13^. In the original analysis it was shown that the proportion of CAR^+^ CD57^+^T-bet^+^CD8^+^ identified at peak day were predictive of outcome, i.e., complete remission (CR) or progressive disease (PD) at 6 months^13^.

**Fig. 7:**
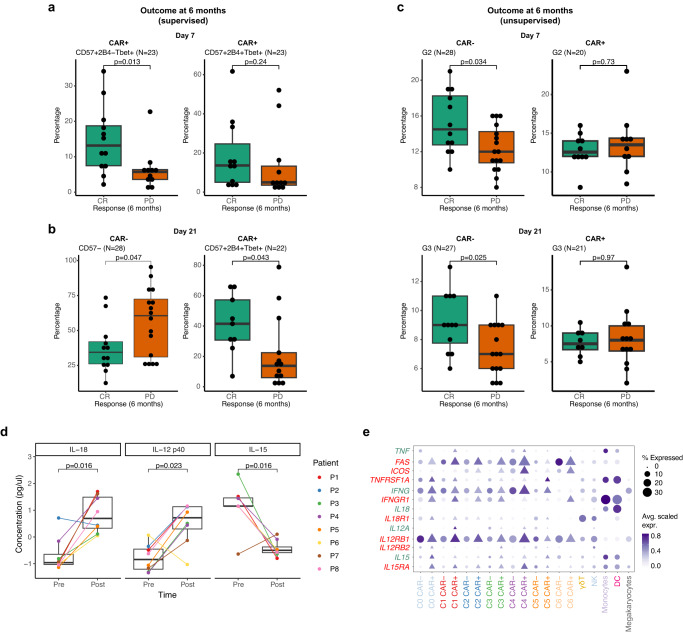
Association with clinical outcomes of patients’ and CAR^+^ CD8^+^ T cells. **a** Proportions of CAR^+^ T cell subsets identified via CD57, T-bet and 2B4 at day 7 in patients with CR or PD at 6 months, as in the original published data (*N* *=* 28). **b** Proportions of CAR^−^ CD57^−^ and of CAR^+^CD57^+^T-bet^+^2B4^+^ T cells at day 21 compared between patients grouped by disease outcome at 6 months. **c** Proportions of CAR^+^ and CAR^−^ T cell subsets identified via unsupervised clustering in patients with CR or PD at 6 months, as in the original published data (*N* *=* 28). Shown are clusters G2 at day 7 (top), and G3 at day 21 (bottom), respectively. **d** Serum cytokine levels from pre- to post-infusion (paired Wilcoxon test, *N* = 28). Colours represent patients. **e** Gene expression levels of interleukins and cytokines along with their corresponding receptors in each cell subsets from the scRNA-seq data of 4 patients in the CARTELL cohort. Box plots in (**a**–**d**) indicate pairwise group comparisons performed with two-sided Wilcoxon Rank Sum Tests and Bonferroni correction for multiple testing. Shown are the median and 75% range quantiles. All the observations are shown as dots. Box plot whiskers extends to the minimum and maximum values, with the box encompassing the interquartile range (IQR), and the centre line indicating the median.

We considered subsets of CD57^+^Tbet^+^CD8^+^ T cells based on the expression of the NK-like markers 2B4^56^, and CD11b, which were both available from the original data, and found that the proportion of CAR^+^ CD57^+^T-bet^+^CD8^+^ T cells lacking expression of 2B4 at day 7 was significantly higher in patients with CR at 6 months, while there was no statistical significance with the 2B4^+^ subset (Fig. 7a). In the late phase, day 21, the subset of CAR^+^ CD57^+^T-bet^+^2B4^−^ T cells was associated with maximum ICANS score (Supplementary Fig. 6b). Notably, at day 21, the proportion of CAR^−^ CD57^−^ T cells were higher in patients with PD outcome at 6 months, while an opposite trend was found for CAR^+^CD57^+^T-bet^+^2B4^+^ subset (Fig. 7b). We also investigated the NK-like subset identified by CD11b, and found that both proportions of CAR^+^ and CAR^−^ T cells expressing CD57, T-bet^+^, and CD11b were increased in patients who achieved CR at 6 months (Supplementary Fig. 6b). NK-like subsets at pre-infusion were higher in patients with negative CMV status, while the opposite association was found for CD57^−^ subsets (Supplementary Fig. 6c).

Further investigation of the CAR^+^ and CAR^−^ T cells, revealed that CAR^−^ T cells from clusters G2 identifying NK-like cells (Fig. 6) were found in higher proportions at day 7 in patients who were associated with CR at 6-month (Fig. 7c). Later, at day 21, a similar association was observed in CAR^−^ T cells from G3 (Fig. 7c). Differential protein expression to identify differences between the CAR^+^ and CAR^−^ T cells from G2 was performed to explain the observed associations with outcome. CAR^−^ T cells from G2 expressed high levels of NK-like markers CD57, 2B4, and CD11b (Fig. 7c). Furthermore, CAR^−^ cells in clusters G2 and G3 at day 7 were also associated with outcome at 3 months, while the proportions of cells in G0 were higher in patients with progressive disease, reflecting their high expression of suppressive markers Helios and PD-1 in this cluster, and in line with the CD4 Treg association reported in the original publication (Supplementary Fig. 6d, e).

Finally, we tested the association between NK-like subsets and variations in serum cytokine levels (*N* *=* 40 cytokines), which were available from pre- and post-infusion samples within the CARTELL cohort. Surprisingly, of the 42 serum cytokines tested, only innate-like pro-inflammatory cytokines IL-18 and IL-12p40 showed a significant increase, and IL-15 levels decreased (*p*-value < 0.05, Fig. 7d), while all the other cytokines did not reveal significant changes. These trends were consistent with the expression of *IL-12RB1* and *IL18R1* in the NK-like clusters, C0 and C1. Notably, *IL-15RA* and *IL-15* were mostly expressed on CAR^−^ T cells in these clusters, which all together support the increase in NK-like phenotype within the CD8^+^ T cells observed post-infusion (Fig. 7e).

The clinical associations identified by this analysis suggest that subsets of CAR^+^ CD57^+^Tbet^+^ cells are associated with clinically relevant parameters. The translational ramifications of our findings warrant further investigation into the role and plasticity of CAR^+^ and CAR^−^ T cells in determining the clinical outcome of CAR19 therapies.

## Discussion

In our study, we used single-cell multi-omics and clonal lineage analyses to demonstrate that CAR^+^ and CAR^−^ T cells irrespective of their sample of origin (infusion product from donor or pre-infusion from patient) share a differentiation trajectory during therapy. We showed that this differentiation process leads to a terminally differentiated state corresponding to a subset of NK-like cells, which we discovered to be observed since the early phase of therapy and in both CAR^+^ and CAR^−^ T cells. Our findings suggest that CAR^+^ T cells respond to additive signals from their CAR domain as well as from sources including homoeostatic proliferation induced by lymphodepletion, TCR-dependent stimulation and the innate-like cytokines, IL-12 and IL-18.

Recently, NK-like CD8^+^ T cells were described in CAR^+^ T cells exposed to persistent stimulation in vitro^23,57,58^ However, we observed that the post-infusion CAR^+^ CD8 T cells from our cohort of donors expressed lower levels of transcription factors *SOX4*, and *ID3*, which were identified as in vitro NK-like signatures^12^. Here we propose that NK-like cells expressed the transcription factors *KLF2*, *KLF3*, and *MYBL1*, which have been also reported as features of cytotoxic, non-dysfunctional subsets in tumours infiltrating T cells^13^. Despite similar differentiation trajectories, CAR^+^ and CAR^−^ T cells revealed distinctive gene and protein signatures, notably within the NK-like subsets. Our data showed that CAR^−^ CD8^+^ T cells undergo greater clonal expansion along the differentiation lineages, likely due to pre-existing memory clones surviving lymphodepletion and antigen stimulation. These cells also showed increased expression of NK-like markers such as CD57 and KLRC1. On the other hand, CAR^+^ T cells displayed higher proliferation, exhaustion, and metabolic activity in response to the high antigen stimulation resulting from the addition of CAR, as previously reported^23,57,58^. Furthermore, circulating CAR^+^ T cells have lower expression of genes encoding suppressor of cytokine signalling proteins, *SOCS1, SOCS2*, and *CISH*, when compared to infusion product and circulating patient’s T cells. These molecules attenuate TCR signalling and control the production and function of NK cells by negative regulation of IL-15^48^, thus, supporting the observation of increased T cell activation and cytotoxicity profile in circulating CAR^+^ T cells.

Our analysis of published dataset from Good et al.^13^ revealed differences in NK-like signatures between CAR^+^ and CAR^−^ T cells. The original work reported association between CAR^+^ T cells with a CD57^+^ profile and clinical outcome. We found that only CAR^+^ T cells with CD57^+^T-bet^+^2B4^−^ profile at day 7 were associated with CR outcome at 6 months. The protein 2B4 has been associated with positive activating signalling of CD8 and NK cells via the intracellular SLAM-associated protein (encoded by *SH2D1A*), while negative signalling may be mediated by the binding of Ewing sarcoma-activated transcript 2 (encoded by *SH2D1B*)^59,60^. Our data showed that CAR^+^ T cells have a reduced NK-like profile compared to CAR^−^, with lower expression of 2B4, despite the expression of *SH2D1A* and *SH2D3A* in most cells (Supplementary Data 1), thus suggesting that CAR^+^ T cells engage a signalling pathway different from that of CAR^−^ T cells. Unsupervised analysis also showed an association of CAR^−^ T cells with outcome at 6 months, with cells in cluster G2 identified as a subset of CD57^+^ cells expressing also CD11b. These results suggest that heterogeneous subsets of NK-like cells may be involved in elimination of tumour cells and involve both CAR^+^ and CAR^−^ T compartments. To further support the association of NK-like subsets with clinical outcomes, we found a significant increase in levels of innate-like interleukins IL-12 and IL-18 following infusion. These cytokines are known to influence NK and CD8 T cell activation and differentiation, and have been already used in enhancing the anti-tumour activity of CAR^+^ T cells by promoting their expansion, activation, cytokine production, and infiltration into the tumour microenvironment^61^. Despite these associations with clinically relevant parameters, further research into NK-like signalling on CAR^+^ T cells may elucidate molecular pathways to control cytotoxicity and improve therapy outcomes.

CAR^+^ and immune cells undergo rapid differentiation and clonal expansion following immune-depletion, which is consistent with previous studies suggesting that homoeostatic signals contribute to proliferation in CD19 CAR^+^ T cells following immune-depletion^62^. Two studies have addressed the clonal expansion of CAR^+^ T cells from the infusion product, and both have found that the majority of large clones are cytotoxic CD8^+^ effector T cells, and with a heterogeneous distribution of cell fates following infusion, including exhaustion^22,23^. Here, we demonstrated that CAR^+^ and CAR^−^ T cells shared a common fate, thus suggesting that CAR^+^ T cell differentiation is driven at least in part by non-CAR-specific signalling. Notably, our data did not reveal a distinct subset of terminally exhausted T cells, rather intermediate values of exhaustion-associated genes. Our data instead revealed that progenitors of exhausted cells were present in both product and circulating CAR^+^ as well as patient’s T cells, and these signatures were found in effector and proliferating clusters. Two previous studies reported that higher proportion of exhausted cells are present 4 weeks post-infusion, which is beyond the time scale considered in our study^22,23^. Notably, Wilson and colleagues proposed that early expression of exhaustion marker TIGIT may delay the onset of exhaustion due to its provision of inhibitory costimulatory signalling.

Clonal expansion may also be influenced by memory recall of T cells specific for opportunistic infections, common during the early phase of lymphopenia in both CAR^+ 9^ and patient’s immune cells^63^. Memory responses to viral infections affect immune reconstitution in the context of both HSCT and cellular therapy^64,65^, which could also impact CAR^+^ T cell proliferation and expansion. Clonal expansion of CAR^−^ CD8^+^ T cells was observed, with several clones matching known TCR sequences against common pathogens. The discovery of a similar trajectory between persisting clones and all other CAR^−^ and CAR^+^ T cells suggests that differentiation trajectories are not significantly influenced by infused CAR^−^ T cells, and immune reconstitution is likely the main driver of this dynamics.

The findings of this analysis of a remarkable similarity in the molecular features of CAR^+^ and CAR^−^ T cells in our donor-derived clinical trial when compared to commercial autologous products suggest that the differentiation process undertaken by CAR^+^ T cells is not significantly affected by the genetic differences between the donor and the recipient prior to infusion. Donor-derived products are considered viable solutions to extend CAR T cell treatments to a broader range of patients, with the additional advantage of simplifying or accelerating manufacturing process^66,67^, and several trials are underway utilising donor-derived products, although there is limited understanding of the associated advantages and risks^68^.

Several limitations are noted. We analysed only 4 of the 8 patients using our single-cell multi-omics approach but followed up with high throughput protein profiling using mass cytometry (CyTOF) for all patients. Therefore, we sought to validate our findings on external cohorts^13^. The serum cytokine levels confirmed an NK-like signature of CAR^+^ and CAR^−^ T cells, for instance with the significant increase of IL-12 and IL-18 from pre- to post-infusion. However, these measurements are only correlative, and the source of these cytokines is unclear. More precise investigation should be conducted to identify how the recipient cells of these cytokine stimuli alter their cytotoxicity profiles and thus contribute to CRS and outcome. Finally, we focused on the TCR repertoire analysis of CAR^+^ and CAR^−^ T cells after infusion and did not investigate the repertoire of the infusion product. It is feasible that a fraction of the infusion product may be CAR^−^, and expansion of these cells may follow the expansion trajectory of pre-existing (recipient-derived) CAR^−^ T cell clones which we have demonstrated to influence the final clinical outcome of CAR T cell therapy. Finally, the clinical associations identified in this study warrant further investigations which need to account for larger cohorts and for variability in CAR^+^ T cell products and manufacturing.

This study gives valuable insights into the differentiation dynamics of both patient’s and CAR^+^ T cells. Our findings provide cellular phenotypes and molecular signatures to identify molecular biomarkers that could potentially be measured from the patient’s blood using CAR^−^ cells. Overall, our findings highlight the potential for continued exploration of the complex interplay between CAR T cells and the immune system, with the goal of improving patient’s clinical outcome.

## Methods

### Subjects and samples

All samples were collected from Westmead Hospital, with written informed consent obtained from all participants in the CARTELL study (ACTRN12617001579381). The relevant ethical approvals related to the CARTELL study are from the NHMRC (4089AU RED HREC/14/WMEAD/332) and the University of New South Wales (reference number HC190012). The study design complied with regulations regarding the use of human study participants and was conducted in accordance with the criteria set by the Declaration of Helsinki. Samples consisted of cryopreserved PBMC isolated by density gradient centrifugation. Permission was obtained from individuals to publish their clinical information in Supplementary Table 1.

### Study design

The purpose of this study was to understand the evolution of CAR^+^ T cells and CAR^−^ immune populations circulating in patients during the initial stage of therapy. To this end, we generated and analysed 20 multi-omic datasets comprised of 11 PBMC populations, 8 CAR^+^ T cell populations and one healthy PBMC reference population (publicly released by 10X Genomics) (Supplementary Table 2). We also compiled mass-cytometry (CyTOF) data which comprised of 36 PBMC samples and 8 infusion products. The de-identified patient information is available in Supplementary Table 1.

### CAR19 T cell product manufacturing

Production of CAR19 T cells was based on our previously described methods^28,69^.

### Cell preparation for scRNA-seq

Cryopreserved cells were thawed and transferred to room temperature RPMI 1640 media (Gibco). Subsequent washes were performed with PBS containing 1% BSA. Manual cell counts were performed with trypan blue and C-Chip haemocytometer slides (NanoEntek). Samples were divided into aliquots containing 1 million cells and designated for either CAR19^+^ or PBMC population analysis. Cells were pelleted by centrifugation and incubated with human Fc Block (BD Pharmingen), except for P1 day 13, P2 day 24, and P2 product. Cells were next stained with a cocktail of oligonucleotide-conjugated antibodies (Supplementary Table 3) for 45 min at 4 °C. For samples to be enriched for CAR19^+^ T cells, additional staining with fluorophore-conjugated antibodies for CD3, CAR19, CD19, CCR7, CD8, PD-1, and CD45RA was performed after 25 min of initial incubation with oligonucleotide-conjugated antibodies followed by three washes following incubation. Samples for CAR19^+^ analysis underwent additional viability staining (Fixable Yellow/7-AAD/propidium iodide [Invitrogen]) according to manufacturer’s instructions prior to sorting. Samples for PBMC analysis proceeded directly to single-cell library preparation. Sorting for the CAR^+^ population (lymphocytes/singlets/live cells/CD19^−^/CD3^+^/CAR19^+^ cells) was performed on a BD FACSAria III (BD Biosciences) at the UNSW Flow Cytometry core facility with single-cell precision into 15 mL centrifuge tubes. Compensation was performed with single stained compensation beads and calculated using the associated FACSDiva software (BD Biosciences). Following sorting, cells were pelleted, and a manual cell count was performed.

### Single-cell library preparation and sequencing

Single-cell library preparation steps using the 10X Genomics platform were performed using Chromium Single Cell 3′ Reagents Kits v2 (10X Genomics, PN-120267) and Chromium Single Cell 3′ Reagents Kits v3 (10X Genomics, PN-1000092). An additional AbSeq PCR1 primer (2 μl) (BD Biosciences, cat. No. 91-1086) was added to the cDNA amplification master mix to enable amplification of the AbSeq oligonucleotide-conjugated antibodies. Following cDNA amplification, single-cell gene expression libraries were purified using SPRIselect beads (0.6×) (Beckman Coulter). The supernatant (containing the AbSeq libraries) was retained, and an additional SPRIselect bead incubation was performed (1.8×). AbSeq libraries were eluted in 25 μl of buffer EB. AbSeq sample index PCR was performed using 11 μl of AbSeq products, 2 μl SI-PCR primer (from 10 × 3′ v2 kit), 2 μl reverse indexing primer (supplied from BD Biosciences), 25 μl 2 × PCR master mix (supplied from BD Biosciences), and 10 μl water. Reactions were incubated: denaturation 98 °C for 45 s, PCR cycling of 98 °C for 20 s, 54 °C for 30 s, 72 °C for 20 s, and final extension 72 °C for 1 min. Between 10 and 15 cycles were used based on input cell numbers. Following sample index PCR, AbSeq libraries were purified with a double-sided SPRIselect bead size selection (0.6× and 1.0×).

Single-cell library preparation steps using the BD Rhapsody platform were performed according to the manufacturer’s protocol for ‘mRNA Whole Transcriptome Analysis (WTA), AbSeq, and Sample Tag Library Preparation’ (Revision 23-21752-00). Single-cell library preparation for CAR T cell products from patients P1, P7, and P8 were performed using the BD Rhapsody platform. All other scRNA-seq libraries were generated on the 10X single-cell platform.

Sequencing libraries were quality controlled using a combination of agarose gel electrophoresis, Quant-iT™ PicoGreen™ dsDNA Assay (Invitrogen) quantification and running on a LabChip GX platform (Perkin Elmer) using a High Sensitivity Assay (CLS760672). Gene expression and AbSeq libraries were pooled and sequenced on a combination of Illumina NextSeq500 and NovaSeq6000 platforms at the UNSW Ramaciotti Centre for Genomics using recommended read lengths.

### Immune receptor sequencing

Full-length TCR and BCR were obtained from 10 × 3′ library via the RAGE-seq methods, as previously described^50^. Briefly, between 50 and 500 ng of full-length cDNA generated from the Chromium Single Cell 3′ protocol (10X Genomics) was used for targeted enrichment of all functional TCR and BCR genes following the Roche-NimbleGen double capture protocol. cDNA libraries were incubated overnight at 47 °C with probes and hybridisation reagents (SeqCap EZ Accessory Kit v2; SeqCap EZ Hybridisation and Wash Kit) and then washed and hybridised a second time overnight for further enrichment. Following each round of hybridisation and capture, PCR was performed using KAPA HotStart HIFI with 1 µM 10X_F primer (AAGCAGTGGTATCAACGCAGAGT) and 1 µM 10X_R primer (CTACACGACGCTCTTCCGATCT) with the following conditions: 98 °C for 3 min; [98 °C for 20 s, 65 °C for 15 s, 72 °C for 1 min 30 s] × 5 cycles (first round) or × 20 cycles (second round); 72 °C for 3 min. Post-capture cDNA library size ranged from 0.6 to 2 kb.

Post-capture cDNA libraries were prepared for long-read sequencing using Oxford Nanopore Technologies’ (ONT) 1D adaptor ligation sequencing kit (SQK-LSK109 or SQK-LSK110) and sequenced on R9.4.1 PromethION flow cells (FLO-PRO002) were base-called using Guppy (v3 or v4). The resulting base-called FASTQ files were de-multiplexed by 10× cell barcodes, using a direct sequence matching strategy described previously^50^. Briefly, cell-barcode sequences (16 nucleotides) were used to demultiplex the nanopore sequencing reads by scanning all reads longer than 250 bases for a matching sequence and allowing for a maximum of 2 mismatches. All reads assigned to a given cell were assembled de novo into contigs using Canu (v1.8), polished using Racon (v1.3.3), and then analysed with IGBlast.

### Pre-processing of single-cell RNA-seq and AbSeq data

Gene expression matrices corresponding to UMI counts from Rhapsody scRNA-seq libraries were produced using the BD Seven Bridge Genomics platform. Matrices from 10× scRNA-seq libraries were produced using the Cell Ranger workflow (v3.0.1, 10X Genomics). We utilised a modified GRCh38 reference genome which was appended with a sequence that corresponded to the transcript product of the CAR T transgene. For the 10X samples, a custom script obtained from the AbSeq manufacturer (BD® bioinformatics scomix@bdscomix.bd.com) was applied to the protein expression base call files to obtain the protein expression matrix. The healthy control sample was downloaded from the 10X website (https://www.10xgenomics.com/resources/datasets).

Samples were quality controlled to remove cells with low mitochondrial content, and low/high number of unique genes and total counts. Gene expression was normalised using scran with default parameters, while protein expression normalised using centred-log-ratio (CLR) normalisation. Potential doublets were removed using the function doubletCluster in the R package scran^70^.

### Clustering and identification of T cell subsets

Dimensionality reduction and clustering for the scRNA-seq dataset was performed using the Seurat R package^71^ (v4.1.0). The top 3000 highly variable genes were extracted using the FindVariableFeatures function. Gene expression matrices was aggregated using the FindIntegrationAnchors and IntegrateData functions. Principal component analysis was run using the RunPCA function with the option npcs = 30 following by neighbour and cluster calculation using the FindNeighbours and FindClusters functions respectively. We explored resolution parameters in the range of 0.01 to 0.6 to determine the marker genes and proteins which adequately explained the observed clusters.

We used these protein and gene markers to classify canonical immune cell types: CD8^+^ T cells (CD8^+^, CD3^+^, CD4^−^, *CD8A*^+^), Tγδ cells (CD3^+^, CD4^−^, CD8^−^, *TRDC*^+^, *TRGC1*^+^), natural killer (NK) and NKT cells (CD8^−^, CD56^high^, CD16^+^, *NKG7*^+^), CD4^+^ T cells (CD4^+^, CD3^+^, CD8^−^), monocytes (CD14^+^, CD3^−^, *CD14*^+^), dendritic cells (CD3^−^, CD14^−^, CD16^−^, *HLA-DRB1*^+^), B cells (CD3^−^, *JCHAIN*^+^, *ITM2C*^+^) and megakaryocytes (*NRGN*^+^).

### Differential expression analysis

Two differential expression analyses were performed depending on whether the analysis was performed within the same sample to find heterogeneous clusters, or between different samples to account for inter-sample differences.

In the first approach, for each sample, differential gene expression (DGE) and differential protein expression (DPE) (intra-sample) analysis were both performed using the FindAllMarkers function, with the “MAST”^72^ and “Wilcoxon sum rank test” test options respectively. Differentially expressed genes and proteins (*p*-adjust < 0.05) that appeared in >50% of samples were designated marker gene and proteins. UMAP plots were used for visualisation using the RunUMAP command with nearest neighbour = 30 and min_dist = 0.01. Dot plots were generated using ggplot2, with average expression calculated across all samples and patients.

In the second approach, DGE and DPE (inter-sample) analysis were performed using the edgeR wrapper run_de function in the Libra R library^73^ with the likelihood-ratio and pseudobulk option, with the patients serving as the replicates.

### Gene set analysis

Gene set enrichment analysis (GSEA) was performed using the fgsea function in the fgsea R package^74^ with default parameters, based on DGE results ranked by the fold-change. Pathways and gene sets were downloaded from the MsigDB^75,76^ in combination with curated T cell specific gene sets (Supplementary Data 5). Average NES values were obtained by averaging over the NES values over each sample. The package Aucell^77^ with default parameters was used to assign gene signature scores.

### Cell-cycle analysis

Cell-cycle scoring and annotation was performed using the CellCycleScoring function in the Seurat package.

### Trajectory analysis

The PAGA plots were generated by running pca (scanpy:pca), finding the nearest neighbours (scanpy:neighbours) and running PAGA (scanpy:paga) using default parameters on the integrated gene expression matrix, and using the pre-defined CD8^+^ clusters.

Slingshot was applied to the UMAP formed from the integrated gene expression matrices using the getLineages and getCurves functions in the Slingshot package^78^, and by manually assigning an initial root value. The pseudotime values generated from Slingshot were used to generate the smoothed gene expression and Aucell vs scaled pseudotime curves, where the smoothed expressions were calculated using the geom_smooth R function with default parameters and the pseudotime was scaled to between 0 and 1 to ensure the endogenous and CAR T pseudotime values corresponded to the same clusters.

### T cell receptor sequencing and analysis

T cell receptor CDR3 amino-acid sequences were extracted using RAGE-seq^50^. Analysis was performed with the bioinformatics pipeline proposed in the original manuscript to identify full-length TCR sequences.

### Identification of known TCR specificities

To identify TCR sequences with known specificity, we downloaded all publicly available, paired TCRαβ CDR3 sequences from the VDJdb database (accessed January 2023)^51^. We then used the Levenshtein distance to measure pairwise distances between TCRαβ CDR3 sequences. A close match was identified by a Levenshtein distance not exceeding 4 (i.e., 4 mismatches) for each of the two sequences, α and β. And including amino-acid differences or insertion/deletions due to different CDR3 length.

### Reference-based clustering

The projection of published (query) datasets onto the clusters identified by our (reference) datasets was performed following the anchor-based transfer method in Seurat (v4.1.0). The FindTransferAnchors function was first used to identify anchors, representing common features between our reference and published query datasets. The TransferData function was then used to transfer the cell type annotations from our reference dataset to the published query dataset.

### Mass cytometry (CyTOF)

Cryopreserved cells were thawed, washed and counted prior to staining for mass cytometry, as was previously reported^28^, using methods that are previously described in detail^64^. First, cells were stained for viability (1.25 µm cisplatin, Fluidigm, San Francisco, CA, USA) staining followed by barcoding of IP and donor PBMCs with anti-CD45 antibodies conjugated to unique mass labels and then incubated with surface metal-conjugated monoclonal antibodies. Cells were then fixed and permeabilised using the FoxP3 buffer kit (eBioscience, San Diego, CA, USA). Finally, cells were incubated with metal-conjugated monoclonal antibodies targeting intracellular proteins and then acquired using with a CyTOF 2 Helios upgraded mass cytometer (Fluidigm). Data was collected in FCS3 format and signal intensity was normalised using EQ Four Element Calibration Beads. Data was analysed using FlowJo (v10.4.2). All antibodies were purchased from Fluidigm and validated by the Ramaciotti Facility for Human Systems Biology Mass Cytometry Reagent Bank, Sydney, Australia. Unconjugated mAbs were purchased and conjugated with metal isotopes using the MaxPar X8 labelling reagent kits (Fluidigm). The list of antibodies was published in Bishop et al.^28^ and are reported in Supplementary Table 4.

Analyses of mass-cytometry data were performed in R using the Seurat package. Cells were gated as CD3^+^CD8^+^CD4^−^ events, and CAR^+^ and CAR^−^ T cells were selected based on CAR expression. Each sample was down-sampled to 3000 cells to achieve reasonable computational runtimes. Protein expression levels were then log-transformed, followed by sample integration using the FindIntegrationAnchors and IntegrateData functions. To identify cluster proteins, DPE analysis was performed using the FindAllMarkers function using the “Wilcoxon sum rank test” test. To identify differential proteins between circulating CAR^+^ and CAR^−^ cells, we used the edgeR wrapper from the run_de function in the Libra R library^73^, with patients serving as replicates. UMAP was used to visualise clustering, with nearest neighbour = 30 and min_dist = 0.1.

For the reanalysis of published CyTOF data, we downloaded the batch-normalised gated T cells as provided by the authors^13^, for which CAR^+^ and CAR^−^ samples were already sorted. We then selected cells gated as CD8^+^CD4^−^ (Supplementary Fig. 6g), with the rest of the analysis following the same pipeline described above for sample integration, clustering, differential protein analysis and UMAP visualisation. The gating strategy for subsets of CD8 T cells used in Fig. 6 were obtained by additional gating of the CD8^+^CD4^−^ cells for CD57, 2B4 and T-bet (Supplementary Fig. 6g).

### Serum cytokines levels

A commercial 42-plex human cytokine Discovery Assay was performed by Eve Technologies on serum samples obtained from each patient before CAR19 T cell infusion and at time points over the first 30 days post-infusion.

### Statistical analysis

Pairwise group comparison analysis was performed using a two-sided paired Wilcoxon test. For the comparisons between >2 groups, correction for multiple testing was performed using the Bonferroni method.

To assess correlation, we calculated Spearman’s rank correlation coefficient.

### Reporting summary

Further information on research design is available in the Nature Portfolio Reporting Summary linked to this article.

### Supplementary information

Supplementary Information

Peer Review File

Description of Additional Supplementary Files

Supplementary Data 1

Supplementary Data 2

Supplementary Data 3

Supplementary Data 4

Supplementary Data 5

Reporting Summary

### Source data

Source Data

## Data Availability

The single-cell gene and protein expression data generated in this study have been deposited in the GEO database under accession code GSE224550 The raw data for the VDJ sequences are available in the SRA under the accession number PRJNA1039523 Further data enquiries (including for the individual de-identified participant data for the patients enrolled in ACTRN12617001579381) should be directed to the corresponding author. The mass cytometry publicly available data used in this study are available in the Stanford Digital Repository [https://purl.stanford.edu/qb215vz6111], and the corresponding clinical data in Supplementary Table S1 of the original study13. The remaining data are within the Article, Supplementary Information or Source Data file. Source data are provided as Source data file. Source data are provided with this paper.

## Code availability

Custom scripts and data required to perform the analyses presented in this study are provided via Zenodo under the link: 10.5281/zenodo.10032198.
