# Supplementary material for: CAR+ and CAR− T cells share a differentiation trajectory into an NK-like subset after CD19 CAR T cell infusion in patients with B cell malignancies
Source: Nat Commun. 2023 Nov 27;14:7767. doi: 10.1038/s41467-023-43656-7 (PMC10682404; doi:10.1038/s41467-023-43656-7)
Supplement: Supplementary file 1 — Supplementary Information [file 41467_2023_43656_MOESM1_ESM.pdf]

SUPPLEMENTARY TABLES

Supplementary Table 1. Patients' clinical information

#	Age	Sex	Disease	Primary refractory	Prior auto	CMV status prior to infusion	Matched sibling allo conditioning	DLI	Prior blin	Total prior lines of therapy	Status at first dose CAR-T	Treated on-trial?	CAR19 T cell doses administered (lymphodepletion)	Best response^ (dose# acheived)	Duration of response (days)	CRS†	CAR T cell malignancy
P1	25	F	B-ALL	No	No	Pos	MAC	No	Yes	5	Extramedullary	Yes	1×10^7 cells/m^2 (Flu/Cy)<br>5×10^7 cells/m^2 (Cy)<br>5×10^7 cells/m^2 (Flu/Cy)<br>1×10^8 cells/m^2 (Flu/Cy)	CR (1)	154	Gr 1	No
P2	66	M	DLBCL	Yes	BEAM	Pos	RIC	3	No	8	Stage IV	Yes	1×10^7 cells/m^2 (Flu/Cy)<br>5×10^7 cells/m^2 (Cy)<br>1×10^8 cells/m^2 (Cy)	PR (1)	335*	No	3.2 months
P3	61	M	B-ALL	No	No	Pos	RIC	No	No	4	MRD +	Yes	1×10^7 cells/m^2 (Flu/Cy)<br>5×10^7 cells/m^2 (Cy)<br>5×10^7 cells/m^2 (Flu/Cy)<br>1×10^8 cells/m^2 (Flu/Cy)	CR (1)	105	No	No
P4	42	M	B-ALL	No	No	Pos	MAC	No	Yes	5	CMR	No (prior seizures)	1×10^7 cells/m^2 (Flu/Cy)<br>5×10^7 cells/m^2 (Flu/Cy)<br>1×10^8 cells/m^2 (Flu/Cy)	CR (1)	230*	No	No
P5	18	M	B-ALL	No, MRD+	No	Neg	MAC	3	Yes	5	Blasts 70%	No (IFI)	1×10^7 cells/m^2 (Flu/Cy)<br>1×10^7 cells/m^2 (Flu/Cy)	CR (1)	224*	Gr 3	No

													5 × 10^7 cells/m^2 (Flu/Cy)				
													1 × 10^8 cells/m^2 (Cy)				
P6	32	M	DLBCL	Yes	No	Pos	N/A	N/A	No	3	Stage III	No	1 × 10^7 cells/m^2 (Flu/Cy)	PR (2)	31	No	No
													5 × 10^7 cells/m^2 (Flu/Cy)				
													38.6x10^6 cells/m^2 (Flu/Cy)				
P7	47	M	B-ALL	No, MRD+	No	Pos	MAC	No	Yes	4	BM: CMR Occult extramedullary	Yes	1 × 10^7 cells/m^2 (Flu/Cy)	CR (1)	125*	Gr 2	No
P8	30	M	B-ALL	No	No	Pos	MAC	No	Yes	5	MRD +	Yes	1 × 10^7 cells/m^2 (Flu/Cy)	CR (1)	97*	No	11.5 months

Supplementary Table 2 Single cell multi-omics data generated in each sample

Patient	Samples	Population	Days	Technologies
P1	Infusion product	CAR T-cells	NA	10x only + Rhapsody/AbSeq + CyTOF
	Circulating	CAR T-cells	13	AbSeq/10x/RageSeq + CyTOF
	Pre-chemo	PBMC	-3	CyTOF
	Pre-infusion	PBMC	-1	AbSeq/10x/RageSeq + CyTOF + Cytokines
	Post-infusion	PBMC	7	AbSeq/10x/RageSeq + Cytokines
	Post-infusion	PBMC	13	AbSeq/10x/RageSeq + CyTOF + Cytokines
	Post-infusion	PBMC	28	CyTOF + Cytokines
P2	Infusion product	CAR T-cells	NA	AbSeq/10x/Ragseq + CyTOF + TCR deep sequencing
	Circulating	CAR T-cells	24	AbSeq/10x + CyTOF + RageSeq
	Pre-chemo	PBMC	-3	CyTOF
	Pre-infusion	PBMC	-1	AbSeq/10x/RageSeq + CyTOF + Cytokines
	Post-infusion	PBMC	24	AbSeq/10x + CyTOF + Cytokines
	Post-infusion	PBMC	31	AbSeq/10x/RageSeq + CyTOF
P3	Infusion product	CAR T-cells	NA	CyTOF
	Circulating	CAR T-cells	14	CyTOF
	Circulating	CAR T-cells	24	CyTOF
	Pre-chemo	PBMC	-3	CyTOF
	Pre-infusion	PBMC	-1	CyTOF+ Cytokine
	Post-infusion	PBMC	14	CyTOF+ Cytokine
	Post-infusion	PBMC	24	CyTOF+ Cytokine (D28)
P4	Infusion product	CAR T-cells	NA	CyTOF
	Circulating	CAR T-cells	16	CyTOF
	Circulating	CAR T-cells	28	CyTOF
	Pre-infusion	PBMC	-1	CyTOF+ Cytokine
	Post-infusion	PBMC	16	CyTOF+ Cytokine

	Post-infusion	PBMC	28	CyTOF+ Cytokine
P5	Infusion product	CAR T-cells	NA	CyTOF
	Circulating	CAR T-cells	11	CyTOF
	Circulating	CAR T-cells	29	CyTOF
	Pre-infusion	PBMC	-1	CyTOF+ Cytokine
	Post-infusion	PBMC	11	CyTOF+ Cytokine (D10)
	Post-infusion	PBMC	29	CyTOF+ Cytokine
A1	Infusion product	CAR T-cells	NA	CyTOF
	Circulating	CAR T-cells	17	CyTOF
	Circulating	CAR T-cells	28	CyTOF
	Pre-infusion	PBMC	-1	CyTOF+ Cytokine
	Post-infusion	PBMC	17	CyTOF+ Cytokine
	Post-infusion	PBMC	28	CyTOF+ Cytokine
P7	Infusion product	CAR T-cells	NA	AbSeq/Rhapsody + CyTOF
	Circulating	CAR T-cells	24	AbSeq/10x/RageSeq+ CyTOF
	Pre-infusion	PBMC	-1	AbSeq/10x/RageSeq + CyTOF + Cytokine
	Post-infusion	PBMC	24	AbSeq/10x/RageSeq + CyTOF + Cytokine
P8	Infusion product	CAR T-cells	NA	AbSeq/Rhapsody + CyTOF + TCR deep sequencing
	Circulating	CAR T-cells	8	AbSeq/10x/RageSeq + CyTOF
	Pre-infusion	PBMC	-1	AbSeq/10x/RageSeq + CyTOF + Cytokine
	Post-infusion	PBMC	8	AbSeq/10x/RageSeq + CyTOF + Cytokine
	Post-infusion	PBMC	21	Abseq/10x
Healthy	Not applicable	PBMC	NA	10x only

Supplementary Table 3. List of oligonucleotide-conjugated and fluoroconjugated antibodies

Target	Clone	Barcode/fluorophore	Catalogue number	Vendor	Conjugate	Dilution
CD127	HIL-7R-M21	AHS0028	940012	BD	Oligonucleotide	1:100
CD14	MϕP9	AHS0037	940005	BD	Oligonucleotide	1:100
CD161	DX12	AHS0002	940070	BD	Oligonucleotide	1:100
CD183	1C6/CXCR3	AHS0031	940030	BD	Oligonucleotide	1:100
CD185 (CXCR5)	RF8B2	AHS0039	940042	BD	Oligonucleotide	1:100
CD19	HIB19	Precommercial: v2a_Hs_0030		BD	Oligonucleotide	1:100
CD194	1G1	AHS0038	940047	BD	Oligonucleotide	1:100
CD196 (CCR6)	11A9	AHS0034	940033	BD	Oligonucleotide	1:100
CD197 (CCR7)	150503	Precommercial: v2a_Hs_007		BD	Oligonucleotide	1:100
CD25	M-A251	Precommercial: v2a_Hs_0026		BD	Oligonucleotide	1:100
CD27	M-T271	AHS0025	940018	BD	Oligonucleotide	1:100
CD28	CD28.2	AHS0024	940017	BD	Oligonucleotide	1:100
CD3	SK7	AHS0033	940000	BD	Oligonucleotide	1:100
CD38	HIT2	AHS0022	940013	BD	Oligonucleotide	1:100
CD4	SK3	AHS0032	940001	BD	Oligonucleotide	1:100
CD45RA	HI100	AHS0009	940011	BD	Oligonucleotide	1:100
CD45RO	UCHL1	AHS0036	940022	BD	Oligonucleotide	1:100
CD8	RPA-T8	AHS0027	940003	BD	Oligonucleotide	1:100
CD95	DX2	AHS0023	940037	BD	Oligonucleotide	1:100
HLA-DR	G46-6	Precommercial: v2a_Hs_0035		BD	Oligonucleotide	1:100
PD1 (CD279)	MIH4	Precommercial: v2a_Hs_0014		BD	Oligonucleotide	1:100
CD10	HI10a	AHS0051	940045	BD	Oligonucleotide	1:100
CD11b	M1/70	AHS0005	940008	BD	Oligonucleotide	1:100

CD11c	B-ly6	AHS0056	940024	BD	Oligonucleotide	1:100
CD137	4B4-1	AHS0003	940055	BD	Oligonucleotide	1:100
CD141	1A4	AHS0083	940079	BD	Oligonucleotide	1:100
CD154	TRAP1	AHS0077	940053	BD	Oligonucleotide	1:100
CD16	3G8	AHS0053	940006	BD	Oligonucleotide	1:100
CD20	2H7	AHS0008	940016	BD	Oligonucleotide	1:100
CD206	19.2	AHS0072	940068	BD	Oligonucleotide	1:100
CD21	B-ly4	AHS0074	940048	BD	Oligonucleotide	1:100
CD274 (B7-H1)	MIH1	AHS0004	940035	BD	Oligonucleotide	1:100
CD40	5C3	AHS0117	940049	BD	Oligonucleotide	1:100
CD56	NCAM16.2	AHS0019	940007	BD	Oligonucleotide	1:100
CD80 (B7-1)	L307.4	AHS0046		BD	Oligonucleotide	1:100
CD86 (B7-2)	2331 (FUN-1)	AHS0057	940025	BD	Oligonucleotide	1:100
IgD	IA6-2	AHS0058	940026	BD	Oligonucleotide	1:100
IgG	G18-145	AHS0059	940027	BD	Oligonucleotide	1:100
LAG-3 (CD223)	T47-530	AHS0018	940080	BD	Oligonucleotide	1:100
TIM-3 (CD366)	7D3	AHS0016	940066	BD	Oligonucleotide	1:100
CD19	SJ25C1	AHS0030	940004	BD	Oligonucleotide	1:100
CD25	2A3	AHS0026	940009	BD	Oligonucleotide	1:100
CD197 (CCR7)	3D12	AHS0007	940014	BD	Oligonucleotide	1:100
PD1 (CD279)	EH12.1	AHS0014	940015	BD	Oligonucleotide	1:100
CD3	UCHT1	BV480	566105	BD	Fluorophore	1:66
CAR19 scFv	136.20.1	AF647	-	B. Jena & L. Cooper, MD Anderson Cancer Center (supplied unconjugated)	Fluorophore	1:50
CD19	HIB19	PE-Cy5	555414	BD	Fluorophore	1:33

Supplementary Table 4. List of antibodies used in mass cytometry

Conjugated label	Specificity	Antibody clone	Manufacturer	Comment
104	CD45	HI30	BD	CAR T product barcode
110	CD45	HI30	BD	Donor PBMC barcode
113	CD56	REA196	Miltenyi	
115	CD8A	RPA-T8	Biolegend	
139	CD57	HCD57	Biolegend	
141	CD49d	9F10	DVS/Fluidigm	
142	CD19	HIB19	BD	
143	CD45RA	HI100	Biolegend	
144	CD69	FN50	Biolegend	
145	CD4	RPA-T4	BD	
146	EOMES	WD1928	Life Technologies	Intracellular
147	CD185 (CXCR5)	RF8B2	BD	
148	CD28	CD28.2	Biolegend	MMM
149	CD366 (TIM3)	7D3	BD	MMM
150	KLRG1	SA231A2	Biolegend	MMM
151	CD39	A1	Biolegend	MMM
152	CD45RO	UCHL1	BD	
153	CD62L	DREG-56	Biolegend	
154	CD196 (CCR6)	REA190	Miltenyi	
155	CD137 (4-1BB)	4B4-1	BD	MMM
156	CD279 (PD1)	EH12.2H7	Biolegend	MMM
158	CD194 (CCR4)	L291H4	Biolegend	
159	CD197 (CCR7)	150503	R&D Systems	
160	CD223 (Lag3)	17B4	Nobus Bio	MMM
161	CD122 (IL-2RB)	TU27	Biolegend	MMM
162	Foxp3	PCH101	eBioscience	Intracellular
163	CD183 (CXCR3)	REA232	Miltenyi	

164	CD274 (PDL1)	MIH1	BD	MMM
165	CD16	3G8	DVS/Fluidigm	
166	TIGIT	MBSA43	RIZO	MMM
167	CAR T	136.20.1	MDACC	
168	Ki67	B56	BD	Intracellular
169	CD25	M-A251	Biolegend	
170	CD3	UCHT1	BD	
171	Granzyme B	GB11	Acris	Intracellular
172	CD38	HIT2	DVS/Fluidigm	
173	Integrin b7	FIB504	BD	
174	HLA-DR	G46-6	BD	
175	Perforin	B-D48	DVS/Fluidigm	Intracellular
176	CD127	A019D5	Biolegend	
209	T-bet	4B10	BD	Intracellular

MMM: Gated using ‘metal minus many’

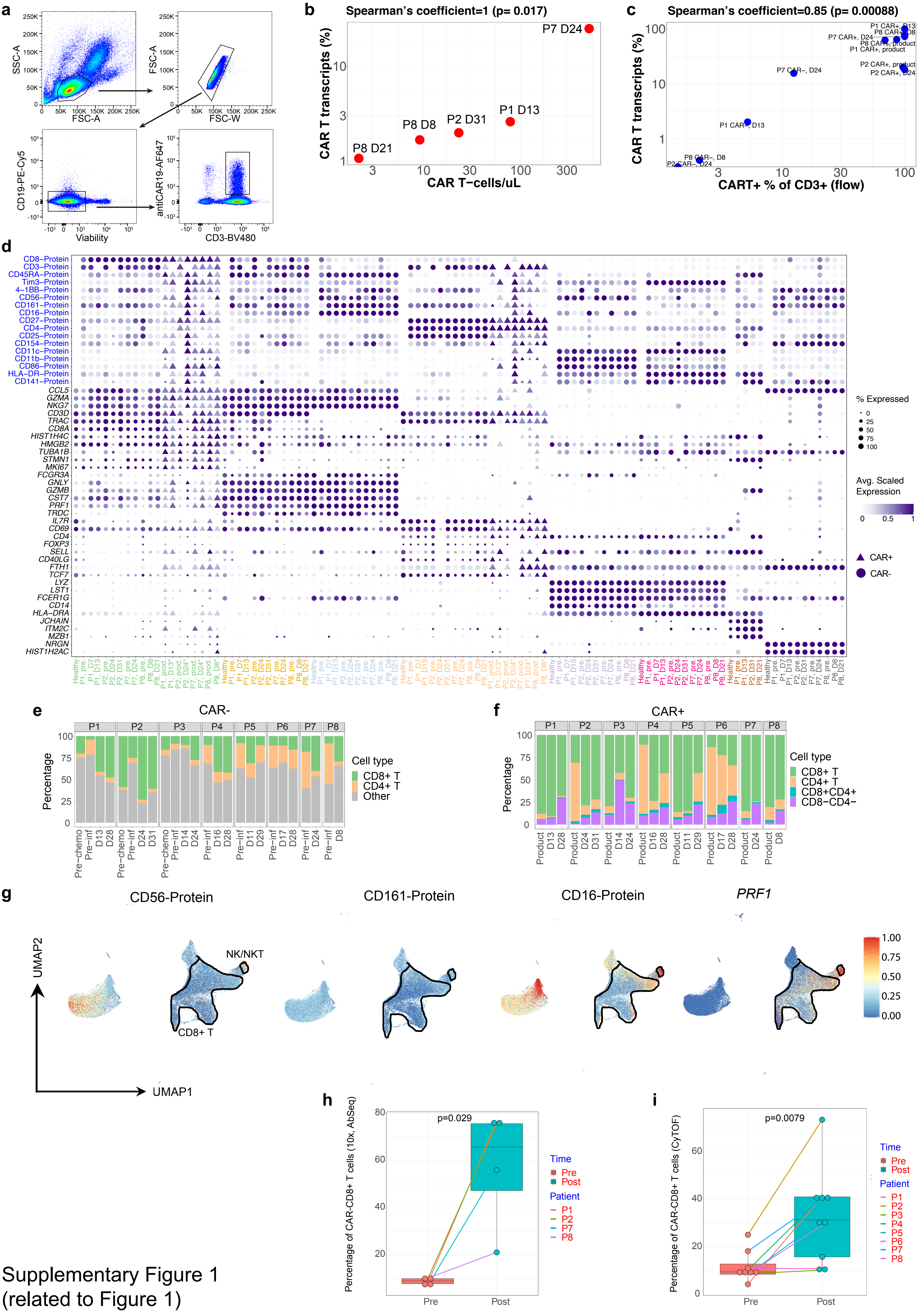

Supplementary Figure 1
(related to Figure 1)

Supplementary Figure 1. Single cell gene and protein expression profiles of patients' immune cells and CAR$^+$ T cells. (a) Gating strategy to identify CAR$^+$ T cells from total PBMC using flow cytometry. (**b-c**) Scatter plots showing the percentage of cells with detectable CAR transcripts versus the number of CAR$^+$ T cell counts in the blood via DNA quantification (**b**), or the frequency of CAR$^+$ T cells sorted using an anti-CAR antibody in flow cytometry **c**). (**d**) Dot plot of selected genes (scRNA-seq) and proteins (AbSeq) for each sample analysed via single cell multi-omics. The size of each point represents the percentage of cells with non-zero expression. The colour represents the avg. (average) scaled log expression. Circles represent CAR$^-$ T cells, triangles represent CAR$^+$ T cells. (**e**) Stacked bar plot of the CD8$^+$ and CD4$^+$ CAR$^+$ T cells in each sample analysed by CyTOF. (**f**) Stacked bar plot of the CD4$^+$ and CD8$^+$ subsets in each CAR$^-$ sample analysed by CyTOF. (**g**) UMAP plots highlighting selected genes and proteins, identifying NK-like CD8$^+$ T cells and unconventional NK and NKT cells. (**h-i**) Proportion of CAR$^+$CD8$^+$ T cells identified with Abseq (N=4 patients) and CyTOF data (N=8 patients), respectively.

Supplementary Figure 2 (related to Figure 2)

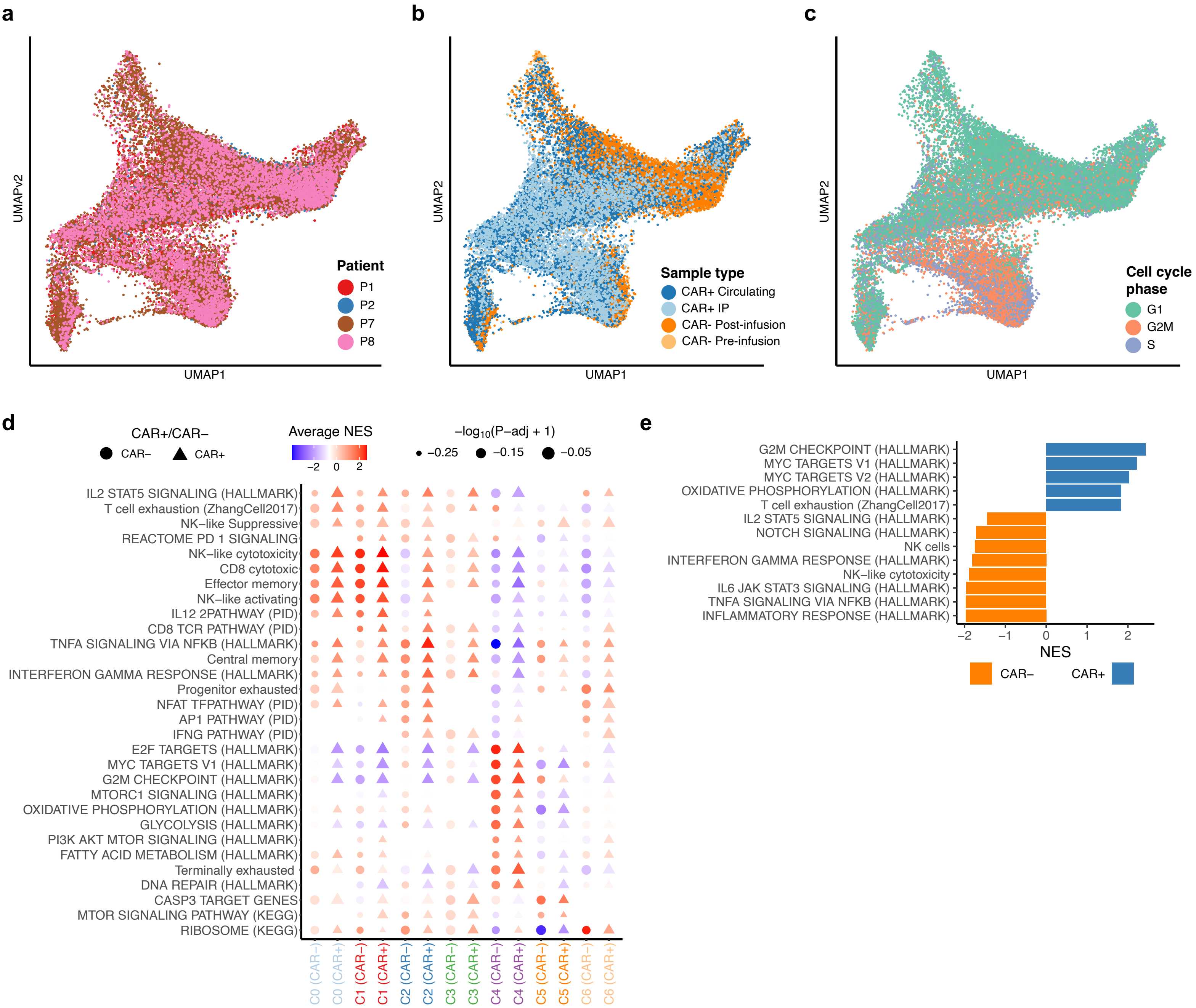

Supplementary Figure 2. Additional characterization of CAR^+ and CAR^- $CD8^+$ T cells in clusters identified using single-cell multi-omics. (a-c) UMAP plots highlighting cell distributions of both CAR^- and CAR^+ T cells according to patients (a), sampling timepoint (b), and of predicted cell cycle phase (c). **(d)** Dot plot of normalised enrichment scores (NES) from the GSEA performed utilising $CD8^+$ cluster genes. Shown are both CAR^- (circles) and CAR^+ (triangles) T cells. Only significant gene sets are shown (adjusted p-value<0.05). The size of each point represents the –log10(p-value). The colour represents the NES. (**e**) Bar plot of NES values from GSEA performed utilising differentially expressed genes identified from the comparison of CAR^- and CAR^+ T cells post-infusion. Gene sets were either taken from the MsigDB database or manually curated (Supplementary Data 5). The full list of GSEA enrichment scores is provided in Supplementary Data 2.

Supplementary Figure 3 (related to Figure 2)

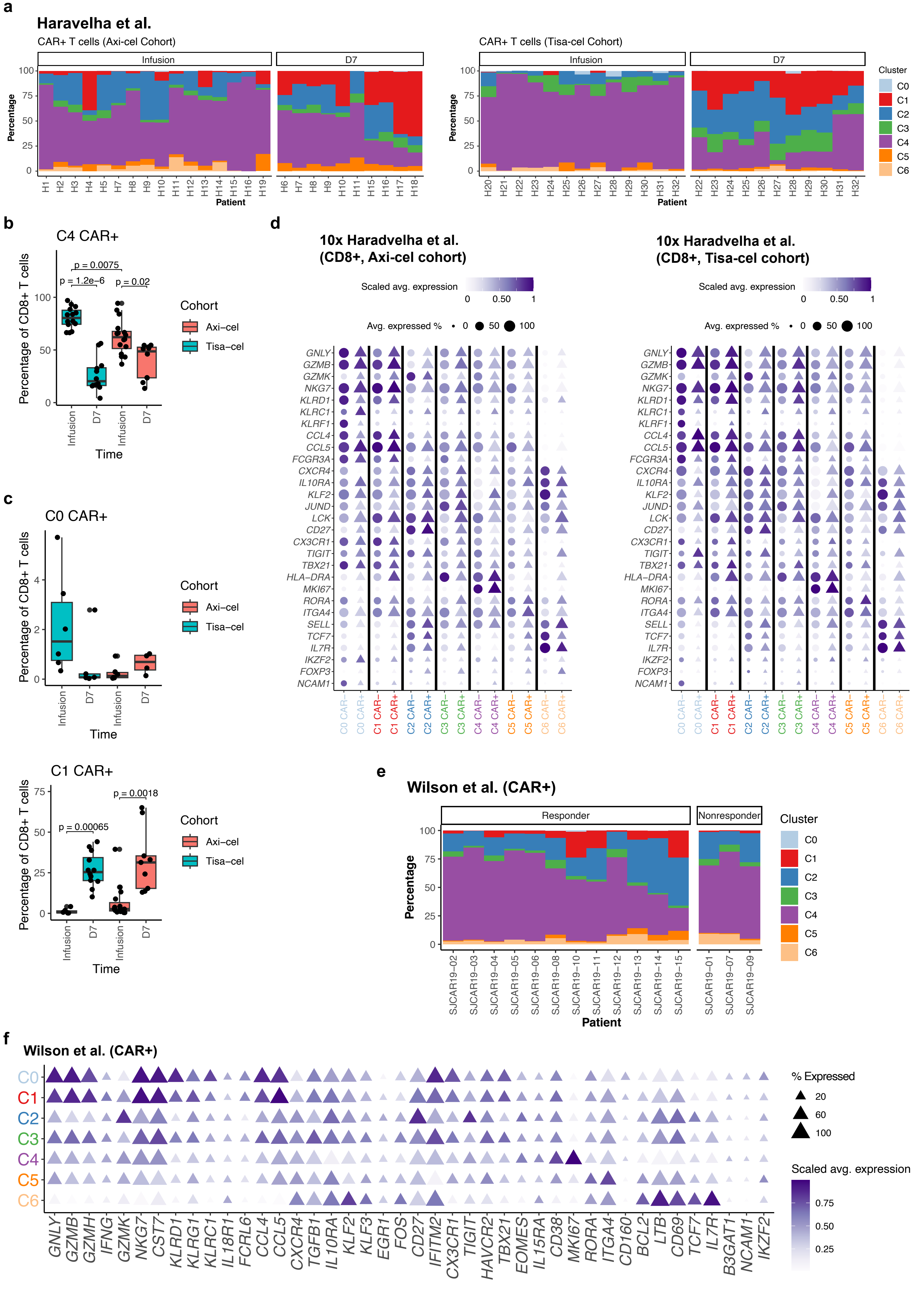

Supplementary Figure 3. Validation of gene and protein expression profiles on published single cell data sets using reference-based clustering. (**a**) Distribution of CAR^+ cells (proportions) in each cluster (referenced based clustering (See Methods for details) using clusters identified with the scRNAseq data from the CARTELL cohort) of the published $CD8^+$ T cell data in Haradhvala et al. [1]. Proportions are split according to cohort type (Axi-cel (*N*=19) or Tisa-cel (*N*=13)) and timepoints (infusion product and D7: Day 7). (**b-c**) Box plots of the percentage of CAR^+CD8^+ T cells in C4 of proliferating cells (b) and of NK-like cells from C0 and C1 (c) across timepoints. Cohorts (Axi-cel and Tisa-cel) are represented with different colours. (**d**) Dot plots of the scaled gene expression values of representative genes of the cluster signatures used as references for the published data split by cohort type (Axi-cel and Tisa-cel). Colours represent gene expression values; the size of each point represents the percentage of cells which express the gene. (**e**) Distribution of cells in the 15 patients from Wilson et al.[2], annotated by $CD8^+$ clusters identified in our data (as per (a)). (**f**) Dot plots of the scaled gene expression values of representative genes of the cluster signatures used as references for the Wilson et al. dataset. Colours represent gene expression values; the size of each point represents the percentage of cells which express the gene.

Supplementary Figure 4 (related to Figure 3)

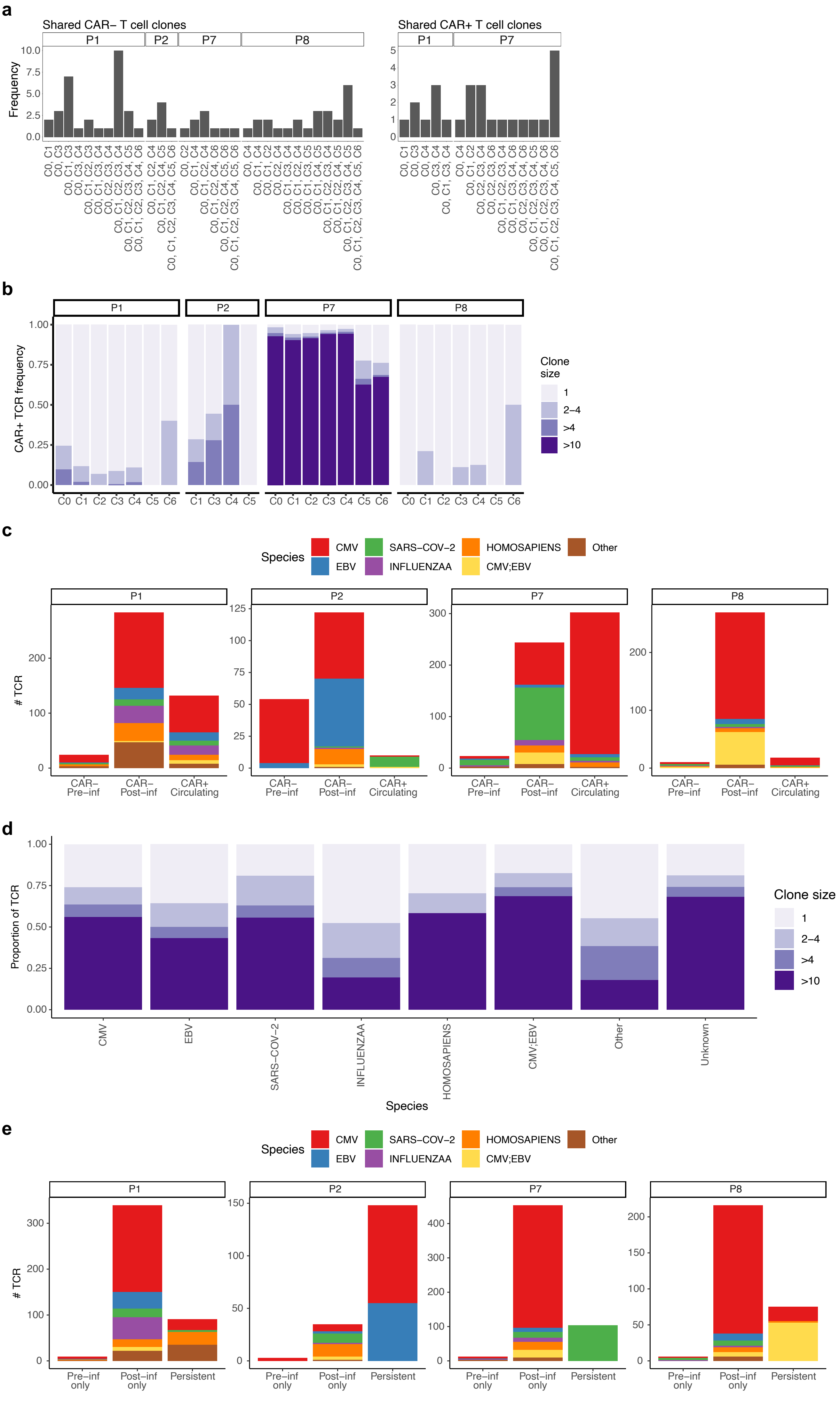

Supplementary Figure 4. Clonal distribution of CAR^- and CAR^+ $CD8^+$ T cells. (**A**) Number of clones shared between C0 and other cluster combinations across both CAR^- and CAR^+ and $CD8^+$ T cells, split by patient. **(b)** Distribution of clones in each cluster, identified by the TCRαβ CDR3 amino acid sequences from the circulating CAR^+CD8^+ T cells. **(c-e)** Distribution of TCRs which had a close or identical match (Levenshtein distance ≤ 3 for each TCR chain) with sequences of known specificity in the VDJdb database (see Methods). (**c**) Each panel represents one patient; each column shows the distribution of clones per sample time point. TCRs are coloured by their epitope species. (**d**) Distribution of clones by their identified specificity against epitope species. (**e**) Distribution of matched TCR sequences from each patient based on whether these were detected in Pre-infusion only, post-infusion only, or persistent between pre and post samples. TCRs are coloured by their epitope species.

Supplementary Figure 5 (related to Figure 4)

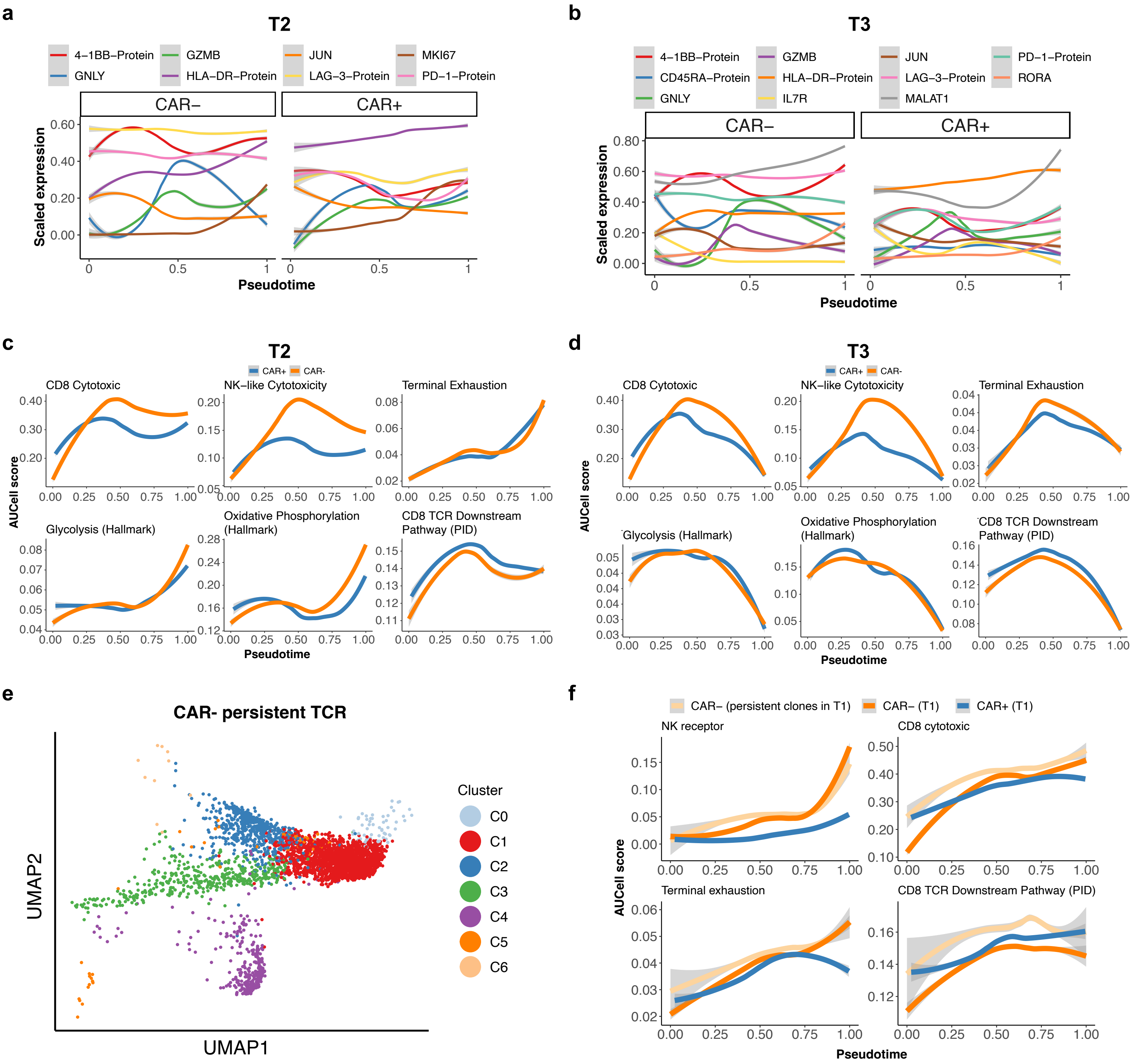

Supplementary Figure 5. Analysis of trajectories T2 and T3 towards proliferating and effector-MAIT cell states and of clonal linages identified from persisting CAR^{-} T cell clones. (a-b) Loess curves obtained from the genes and proteins expression values along the inferred pseudotime values for trajectory T2 (proliferating activating cell state) and T3 (effector-MAIT), respectively. Gray shade represents 95% confidence interval. (**c-d**) Loess curves along T2 and T3, using gene module scores calculated with AUCell versus the scaled pseudotime values. (**e**) UMAP plot highlighting the distribution of persisting clones within CAR^{-} T cells. These clones are identified by TCR sequences found in both pre- and post-infusion samples (**f**) Loess curves along trajectory T1 (NK-like) for CAR^{+}, total CAR^{-} and for CAR^{-} cells forming clonal lineages (shared clonotypes).Trajectory analysis for clonal lineages are identified from the clones with a TCR sequence identified in both pre- and post-infusion samples.

Supplementary Figure 6 (related to Figure 6)

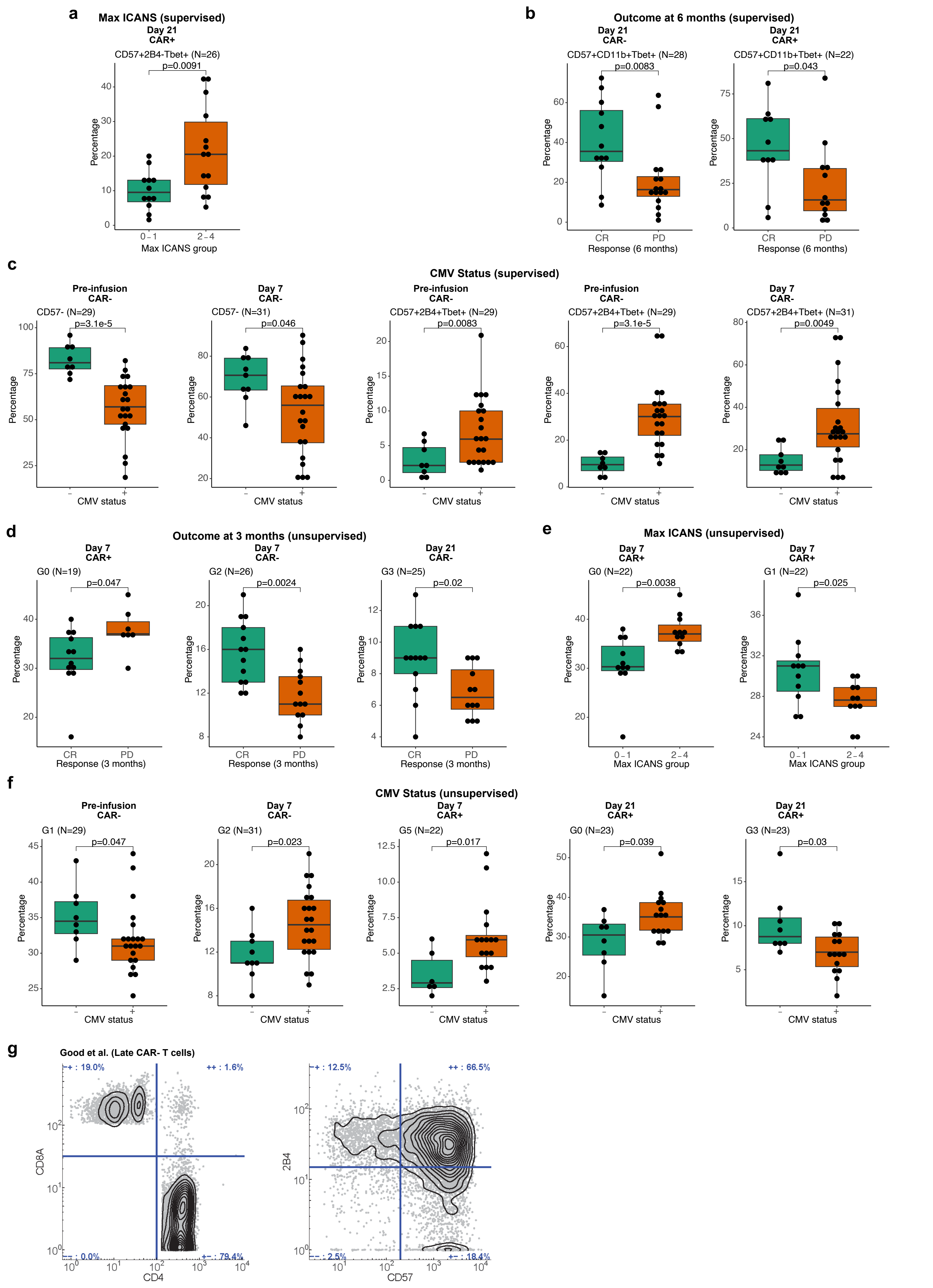

Supplementary Figure 6. Correlation analysis between clinical outcomes and proportion of CAR$^+$ and CAR$^-$ CD8$^+$ T cell subsets. (**a)** Proportions of CAR$^+$ T cell subsets identified via CD57$^+$T-bet$^+$ 2B4$^-$ at day 21 in patients grouped by max ICANS score. (**b**) Proportions of T cell subsets identified via CD57, T-bet and CD11b at day 21 in patients with CR or PD at 6 months, as in the original published data (*N*=28). **(c)**. Proportions of CAR$^-$ T cells in subsets based on the expression of CD57, 2B4 and T-bet at various sample time points as indicated in the legend, with data patients grouped by CMV status. (**d**) Proportions of T cells at day 7 (peak) and day 21 (late) identified using unsupervised clustering in patients with CR or PD at 3 months (*N*=28). Box plots in (a-d) indicate Pairwise group comparisons performed with two-sided Wilcoxon Rank Sum Tests. Shown are the median and 75% range quantiles. All the observations are shown as dots. (**e-f**) Proportions of T cells identified using unsupervised clustering at various sample time points and clusters, with data patients grouped by Max ICANS score (**e**), and by CMV status (**f**). (**g**) Example of gating for CD57 and 2B4 subsets within CD8$^+$ T cells.

References

1. Haradhvala, N.J.*, et al.* Distinct cellular dynamics associated with response to CAR-T therapy for refractory B cell lymphoma. *Nat Med* **28**, 1848-1859 (2022).
2. Wilson, T.L.*, et al.* Common Trajectories of Highly Effective CD19-Specific CAR T Cells Identified by Endogenous T-cell Receptor Lineages. *Cancer Discov* **12**, 2098-2119 (2022).
