# Supplementary material for: CAR+ and CAR− T cells share a differentiation trajectory into an NK-like subset after CD19 CAR T cell infusion in patients with B cell malignancies
Source: Nat Commun. 2023 Nov 27;14:7767. doi: 10.1038/s41467-023-43656-7 (PMC10682404; doi:10.1038/s41467-023-43656-7)
Supplement: Supplementary file 3 — Description of Additional Supplementary Files [file 41467_2023_43656_MOESM3_ESM.pdf]

Description of Additional Supplementary Files

Supplementary Data 1. Differentially expressed genes for all the group comparisons performed in this study.
Supplementary Data 2. Gene set enrichment analysis of differentially expressed genes.
Supplementary Data 3. TCR sequences of CD8+ T cells (CAR- and CAR+ subsets).
Supplementary Data 4. TCR sequences interrogated with VDJdb for known antigen specificity.
Supplementary Data 5. List of curated gene signatures utilised to calculate gene modules' scores.
