# Supplementary material for: CAR+ and CAR− T cells share a differentiation trajectory into an NK-like subset after CD19 CAR T cell infusion in patients with B cell malignancies
Source: Nat Commun. 2023 Nov 27;14:7767. doi: 10.1038/s41467-023-43656-7 (PMC10682404; doi:10.1038/s41467-023-43656-7)
Supplement: Supplementary file 9 — Reporting Summary [file 41467_2023_43656_MOESM9_ESM.pdf]

nature portfolio

Corresponding author(s): Fabio Luciani

Last updated by author(s): Oct 10, 2023

Reporting Summary

Nature Portfolio wishes to improve the reproducibility of the work that we publish. This form provides structure for consistency and transparency in reporting. For further information on Nature Portfolio policies, see our Editorial Policies and the Editorial Policy Checklist.

Statistics

For all statistical analyses, confirm that the following items are present in the figure legend, table legend, main text, or Methods section.

n/a	Confirmed	
☐	☒	The exact sample size (*n*) for each experimental group/condition, given as a discrete number and unit of measurement
☐	☒	A statement on whether measurements were taken from distinct samples or whether the same sample was measured repeatedly
☐	☒	The statistical test(s) used AND whether they are one- or two-sided<br>*Only common tests should be described solely by name; describe more complex techniques in the Methods section.*
☒	☐	A description of all covariates tested
☐	☒	A description of any assumptions or corrections, such as tests of normality and adjustment for multiple comparisons
☐	☒	A full description of the statistical parameters including central tendency (e.g. means) or other basic estimates (e.g. regression coefficient) AND variation (e.g. standard deviation) or associated estimates of uncertainty (e.g. confidence intervals)
☐	☒	For null hypothesis testing, the test statistic (e.g. *F*, *t*, *r*) with confidence intervals, effect sizes, degrees of freedom and *P* value noted<br>*Give P values as exact values whenever suitable.*
☒	☐	For Bayesian analysis, information on the choice of priors and Markov chain Monte Carlo settings
☒	☐	For hierarchical and complex designs, identification of the appropriate level for tests and full reporting of outcomes
☒	☐	Estimates of effect sizes (e.g. Cohen's *d*, Pearson's *r*), indicating how they were calculated

Our web collection on statistics for biologists contains articles on many of the points above.

Software and code

Policy information about availability of computer code

Data collection	Flow cytometry data acquisition and sorting were performed on a BD FACS Aria III.<br>Single-cell library preparation was performed using the Chromium platform from 10X Genomics (3' chemistry)<br>Single-cell sequencing was performed using paired-end Illumina sequencing (NextSeq 500 and NovaSeq 6000) at the UNSW Ramaciotti Centre for Genomics.<br>T-cell receptor sequencing was performed on an Oxford Nanopore Technologies PromethION using 1D adapter ligation sequencing kits (SQK-LSK109 or SQK-LSK110) at the Garvan Institute Sequencing platform.<br>Mass cytometry data were acquired on a CyTOF2 Helios platform.
Data analysis	Single-cell sequencing outputs were processed using CellRanger (10X Genomics) and the Seven Bridge Genomics platform (BD).<br>Base calling for nanopore sequencing output was performed using Guppy (versions 3 and 4). FASTQ files were demultiplexed by matching 10X cell barcodes with barcodes from the single-cell sequencing output. Assigned reads were assembled using Canu (version 1.8), polished using Racon (version 1.3.3) and analysed using IGBlast.<br>Flow cytometry data were analysed using FlowJo (BD, version 10.4.2).<br>Data analyses were performed using R (4.1.2), RStudio (2022.02.1), and Python (3.10).<br><br>R package: R package seurat (version 4.1.0) was used for integration, unsupervised clustering, intra-sample differential gene and protein analysis. ggplot2 (3.3.5) was used for data visualization. Differential gene expression (DGE) and differential protein expression (DPE) (intra-sample) analysis were both performed using the FindAllMarkers function, with the "MAST" and "Wilcoxon sum rank test" test options respectively.

DGE and DPE (inter-sample) analysis were performed using the edgeR wrapper run_de function in the Libra R library with the likelihood-ratio and pseudobulk option, with the patients serving as the replicates.

Gene set enrichment analysis (GSEA) was performed using the fgsea function in the fgsea R package with default parameters, based on DGE results ranked by the fold-change.

The PAGA plots were generated by running pea (scanpy:pca), finding the nearest neighbours (scanpy:neighbours) and running PAGA (scanpy:paga) using default parameters on the integrated gene expression matrix, and using the pre-defined CDS+ clusters. Slingshot was applied to the UMAP formed from the integrated gene expression matrices using the getlineages and getCurves functions in the Slingshot package.

Analyses of mass cytometry data were performed in R using the Seurat package. Protein expression levels were then log-transformed, followed by sample integration using the FindIntegrationAnchors and IntegrateData functions.

Pairwise group comparison analysis was performed using a two-sided paired Wilcoxon test. For the comparisons between >2 groups, correction for multiple testing was performed using the Bonferroni method. To assess correlation, we calculated Spearman's rank correlation coefficient.

For manuscripts utilizing custom algorithms or software that are central to the research but not yet described in published literature, software must be made available to editors and reviewers. We strongly encourage code deposition in a community repository (e.g. GitHub). See the Nature Portfolio guidelines for submitting code & software for further information.

Data

Policy information about availability of data

All manuscripts must include a data availability statement. This statement should provide the following information, where applicable:

- Accession codes, unique identifiers, or web links for publicly available datasets
- A description of any restrictions on data availability
- For clinical datasets or third party data, please ensure that the statement adheres to our policy

All the single cell data of gene and protein expression are deposited with GEO under GSE224550. The single-cell gene and protein expression data generated in this study have been deposited in the GEO database under accession code GSE224550 [https://www.ncbi.nlm.nih.gov/geo/query/acc.cgi?acc=GSE224550]. The raw data for the VDJ sequences are available in the SRA under the accession number PRJNA1039523 [https://www.ncbi.nlm.nih.gov/bioproject/PRJNA1039523/]. Further data enquiries (including for the individual de-identified participant data for the patients enrolled in ACTRN12617001579381) should be directed to the corresponding author.

Research involving human participants, their data, or biological material

Policy information about studies with human participants or human data. See also policy information about sex, gender (identity/presentation), and sexual orientation and race, ethnicity and racism.

Reporting on sex and gender	Sex was reported with consent from the patients. Sex was not considered in the study design, which was based on recruiting a small number of patients (max 20) for a phase I clinical trial addressing safety of an investigator driven CAR product. Details of the cohort is provided in the original publication reporting the results of this clinical trial (Micklethwaite et al. Blood 2021 https://doi.org/10.1182/blood.2021010858. Bishop et al. Blood 2021, 10.1182/blood.2021010813).
Reporting on race, ethnicity, or other socially relevant groupings	We did not consider race, ethnicity of other socially relevant groupings in our study.
Population characteristics	We studied a cohort of patients with relapsed or refractory CD19+ B-cell malignancy following HLA-matched sibling donor allogeneic haemopoietic stem cell transplant.<br><br>Trial eligibility criteria were: relapsed/refractory CD19+ disease after a matched related HSCT performed for lymphoid malignancy; life expectancy 26 weeks; Karnofsky/Lansky score ≥50%, or ECOG ≤2; previous HSCT donor available for the generation of CAR19 Tcells; and use of effective contraception.<br><br>Trial exclusion criteria were: uncontrolled infection; graft versus host disease (GVHD) ≥grade II; donor lymphocyte infusion <10 weeks prior; >0.5 mg/kg of oral prednisolone or equivalent; bilirubin >2x upper limit of normal (ULN); AST >3x ULN; creatinine >2x ULN for age; pulse oximetry ≤90% on room air; pregnant or lactating; past hypersensitivity to murine proteins; and prior seizures.<br><br>Population characteristics were detailed in previous publications and in Supplementary Table 1. Conclusions were not drawn on the basis of characteristics of individual participants. The effect of co-variates was not considered given the small cohort size and highly individualised participant characteristics.
Recruitment	Patients were previously recruited for a clinical trial (ACTRN12617001579381). All patients provided informed consent and did not received any compensation.
Ethics oversight	The trial was approved by the Western Sydney Local Health District Human Research Ethics Committee, Sydney Australia.

Note that full information on the approval of the study protocol must also be provided in the manuscript.

Field-specific reporting

Please select the one below that is the best fit for your research. If you are not sure, read the appropriate sections before making your selection.

- [x] Life sciences
- [ ] Behavioural & social sciences
- [ ] Ecological, evolutionary & environmental sciences

For a reference copy of the document with all sections, see nature.com/documents/nr-reporting-summary-flat.pdf

Life sciences study design

All studies must disclose on these points even when the disclosure is negative.

Sample size	We analysed 7 of the 10 patients enrolled in the clinical study and an additional patient was considered who was treated outside the clinical trial with the same CAR T cells. Individuals were randomly selected from this previous study.<br>No formal sample size power analysis was performed.
Data exclusions	No data were excluded for this study
Replication	We performed no formal replication experiments. We included additional data related to mass cytometry experiments to complement findings identified from the single-cell multi-omics experiments.
Randomization	All participants received CAR T therapy. All Participants belonged to a single treatment group and randomisation was not performed.
Blinding	All participants received CAR T therapy. Investigators were not blinded as all participants received treatment.

Reporting for specific materials, systems and methods

We require information from authors about some types of materials, experimental systems and methods used in many studies. Here, indicate whether each material, system or method listed is relevant to your study. If you are not sure if a list item applies to your research, read the appropriate section before selecting a response.

Materials & experimental systems

n/a	Involved in the study	
[ ]	[x]	Antibodies
[x]	[ ]	Eukaryotic cell lines
[x]	[ ]	Palaeontology and archaeology
[x]	[ ]	Animals and other organisms
[ ]	[x]	Clinical data
[ ]	[x]	Dual use research of concern
[x]	[ ]	Plants

Methods

n/a	Involved in the study	
[x]	[ ]	ChIP-seq
[ ]	[x]	Flow cytometry
[x]	[ ]	MRI-based neuroimaging

Antibodies

Antibodies used

All antibodies sourced from BD unless stated otherwise.

Flow cytometry: CD3-BV480 (clone UCHT1, cat. 566105), CAR19 scFv-AF647 (custom conjugate, unconjugated antibody supplied from B. Jena & L. Cooper, MD Anderson Cancer Center), CD19-PE-Cy5 (clone HIB19, cat. 555414)

Single-cell sequencing:
CD127, cloneHIL-7R-M21
CD14, cloneMφP9
CD161, cloneDX12
CD183, clone1C6/CXCR3
CD185 (CXCR5), cloneRF8B2
CD19, cloneHIB19
CD194, clone1G1
CD196 (CCR6), clone11A9
CD197 (CCR7), clone150503
CD25, cloneM-A251
CD27, cloneM-T271
CD28, cloneCD28.2
CD3, cloneSK7

CD38, cloneHIT2
CD4, cloneSK3
CD45RA, cloneHI100
CD45RO, cloneUCHL1
CD8, cloneRPA-T8
CD95, cloneDX2
HLA-DR, cloneG46-6
PD1 (CD279), cloneMIH4
CD10, cloneHI10a
CD11b, cloneM1/70
CD11c, cloneB-ly6
CD137, clone4B4-1
CD141, clone1A4
CD154, cloneTRAP1
CD16, clone3G8
CD20, clone2H7
CD206, clone19.2
CD21, cloneB-ly4
CD274 (B7-H1), cloneMIH1
CD40, clone5C3
CD56, cloneNCAM16.2
CD80 (B7-1), cloneL307.4
CD86 (B7-2), clone2331 (FUN-1)
IgD, cloneIA6-2
IgG, cloneG18-145
LAG-3 (CD223), cloneT47-530
TIM-3 (CD366), clone7D3
CD19, cloneSJ25C1
CD25, clone2A3
CD197 (CCR7), clone3D12
PD1 (CD279), cloneEH12.1

Validation

Validation of the custom conjugated anti-CAR19 scFv-AF647 antibody was validated by staining with known CAR19 expressing and non-expressing samples. All other antibodies were commercially available products and subjected to routine testing by the supplying vendor.

Clinical data

Policy information about clinical studies
All manuscripts should comply with the ICMJEguidelines for publication of clinical research and a completedCONSORT checklist must be included with all submissions.

Clinical trial registration

This trial was registered at www.anzctr.org.au as ACTRN12617001579381

Study protocol

The study protocol is available on request of the Principal Investigator. Dr Kenneth Micklethwaite- kenneth.micklethwaite@sydney.edu.au

Data collection

Patients were recruited from January 2018 through to the voluntary halting of the trial in August 2019. Data collection for long term safety assessment is ongoing.

Outcomes

The Primary Objective was to evaluate the safety of PiggyBac generated, donor derived allogeneic T-cells that were genetically modified to express chimeric antigen receptors targeting the CD19 molecule in patients with relapsed and persistent B-cell malignancies post allogeneic stem cell transplant.
Infusion and followup were as described by Bishop et al Blood 2021.

Plants

Seed stocks

Report on the source of all seed stocks or other plant material used. If applicable, state the seed stock centre and catalogue number. If plant specimens were collected from the field, describe the collection location, date and sampling procedures.

Novel plant genotypes

Describe the methods by which all novel plant genotypes were produced. This includes those generated by transgenic approaches, gene editing, chemical/radiation-based mutagenesis and hybridization. For transgenic lines, describe the transformation method, the number of independent lines analyzed and the generation upon which experiments were performed. For gene-edited lines, describe the editor used, the endogenous sequence targeted for editing, the targeting guide RNA sequence (if applicable) and how the editor was applied.

Authentication

Describe any authentication procedures for each seed stock used or novel genotype generated. Describe any experiments used to assess the effect of a mutation and, where applicable, how potential secondary effects (e.g. second site T-DNA insertions, mosiacism, off-target gene editing) were examined.

Flow Cytometry

Plots

Confirm that:

☒ The axis labels state the marker and fluorochrome used (e.g. CD4-FITC).

☒ The axis scales are clearly visible. Include numbers along axes only for bottom left plot of group (a 'group' is an analysis of identical markers).

☒ All plots are contour plots with outliers or pseudocolor plots.

☒ A numerical value for number of cells or percentage (with statistics) is provided.

Methodology

Sample preparation

Cryopreserved PBMC and cart cell infusion products

Instrument

BD FACS Aria III

Software

Flowjo version 10

Cell population abundance

Target populations were sorted with single-cell precision.

Gating strategy

A gating strategy has been provided in extended data figure 1. Lymphocytes were selected by forward and side scatter. Sing cells were gated and non viable and CD19+ cells were excluded. The target population of cart cells for sorting was identified by costaining for CD3 and anti-CAR19.

☒ Tick this box to confirm that a figure exemplifying the gating strategy is provided in the Supplementary Information.
